# Source-sink relationship in *Brassica carinata* derived *Brassica juncea* introgression lines for improving water use efficiency under moisture deficit stress conditions

**DOI:** 10.3389/fpls.2026.1816052

**Published:** 2026-05-26

**Authors:** Omkar Maharudra Limbalkar, Prashant Vasisth, Gokulan Dhanasekaran, Mohit Sharma, Mohan Lal Meena, Anshul Watts, Viswanathan Chinnusamy, Naveen Singh

**Affiliations:** 1 Division of Genetics, ICAR- Indian Agricultural Research Institute, New Delhi, India; 2 ICAR-National Institute for Plant Biotechnology, New Delhi, India; 3 Division of Plant Physiology, ICAR-Indian Agricultural Research Institute, New Delhi, India

**Keywords:** abiotic stress, drought tolerance, Ethiopian mustard, Indian mustard, interspecific hybridization, WUE

## Abstract

Improving water-use efficiency and seed yield stability under moisture deficit stress is a major challenge in *Brassica juncea* cultivation. The present study evaluated *Brassica carinata*–derived *B. juncea* introgression lines (ILs) to elucidate the role of source–sink relationships in enhancing seed yield under moisture deficit stress conditions. A set of 191 ILs, along with their parental lines, was assessed under rainfed and irrigated conditions for various agromorphological and physiological traits. Results revealed that ILs consistently outperformed parents for dry matter accumulation at maturity, seed yield per plant, harvest index, flowering duration, and reproductive-stage dry matter accumulation across environments. Under moisture-deficit stress, ILs maintained comparable crop growth rate and net assimilation rate to those of parents, while exhibiting enhanced physiological plasticity under irrigated conditions. Trait association and scatterplot analyses revealed that balanced source–sink ratio and efficient dry matter partitioning in ILs were critical determinants of seed yield under rainfed conditions. Principal component analysis further demonstrated strong multivariate associations of seed yield with crop growth rate, net assimilation rate, leaf area index, reproductive dry matter accumulation, and source–sink traits, clearly differentiating superior ILs from their parents. Several introgression lines, including IL11, IL33, IL54, IL73, IL83, IL112, IL124, IL128, IL134, IL135, IL155, IL161, and IL165, emerged as promising for moisture deficit stress conditions. Overall, the study highlights that integrating physiological efficiency with source–sink traits provides a robust framework for improving drought resilience and yield stability in *Brassica juncea* breeding programs through interspecific hybridization.

## 1 Introduction

The rapeseed-mustard group of crops comprises the most important oilseed crops and is cultivated worldwide. These crops are primarily grown for edible oil, which is rich in unsaturated fatty acids, including omega-3 and omega-6 fatty acids, making it suitable for human consumption and industrial applications (Sachan et al., 2024). Globally, rapeseed-mustard is the second-largest group of oilseed crops after soybean, occupying an area of approximately 42.95 million hectares (mha) and producing about 89.86 million metric tons (MMT) in 2023–24 (Anonymous, 2025). The group comprises six cultivated species, viz., *Brassica rapa* L. (AA, 2n = 2x = 20), *B. nigra* L. (BB, 2n = 2x = 16), *B. oleracea* L. (CC, 2n = 2x = 18), *B. juncea* (L.) Czern & Coss (AABB, 2n = 4x = 36), *B. napus* L. (AACC, 2n = 4x = 38), and *B. carinata* A. Braun (BBCC, 2n = 4x = 34). Among these, three amphidiploids, viz., *B. juncea*, *B. napus*, and *B. carinata*—along with *B. rapa* are predominantly cultivated for edible oil (Gupta and Pratap, 2007). India ranks as the third-largest producer of rapeseed-mustard after Canada and China, with production of 11.6 MMT from 9.25 mha during 2023–24 (Anonymous, 2025). This accounts for approximately 30% of the country’s total oilseed production, highlighting its crucial role in India’s edible oil economy and food security.

Among the rapeseed mustard group of crops, Indian mustard, *Brassica juncea*, is the most extensively cultivated species in India, contributing over 90% of the total acreage in the country. Despite several efforts, mustard productivity remains stagnant, largely due to the crop’s vulnerability to abiotic stresses such as drought and a narrow genetic base arising from intensive breeding with a limited set of elite cultivars, particularly Varuna. According to the Solvent Extractors’ Association of India, the country imported 15.96 MMT of edible oil, valued at over 16 billion dollars, accounting for more than 60% of the country’s total edible oil demand in the year 2023-24 (Source: https://seaofindia.com/). The rise in per capita oil consumption is due to growing population, increasing income and changing dietary preferences, and is expected to increase further (Dalei and Gupta, 2019). To meet the edible oil demand of a growing population, approximately 16.4-20.5 MMT of rapeseed-mustard needs to be produced (DRMR, 2011; Valiyaveettil et al., 2023), whereas current production stands at only around 11.6 MMT (Anonymous, 2025).

To achieve self-sufficiency in edible oil, there is an urgent need to improve the productivity of the Indian mustard. However, limited genetic variability in this species restricts the potential for its improvement (Chauhan et al., 2011). Moreover, Indian mustard is highly vulnerable to a range of biotic and abiotic stresses, resulting in unstable yields across growing seasons. Among abiotic stresses, drought is particularly detrimental and significantly limits the growth and productivity of crop plants (Sehgal et al., 2019; Rasheed et al., 2020). Notably, nearly 24% of its cultivation is under rainfed conditions, where moisture deficit stress leads to considerable yield losses (Chauhan et al., 2007). Most of the present-day varieties of mustard lack resilience to drought, making them unsuitable for regions frequently facing water deficit stress. Although few drought-tolerant cultivars have been developed, however, their limited yield potential has curtailed widespread adoption. Thus, enhancing yield stability under adverse climatic conditions becomes essential for achieving self-sufficiency in edible oilseed production. Addressing this challenge requires a better understanding of the physiological processes that govern biomass production, partitioning, and resource utilization—particularly the source–sink relationship under drought stress (Sadras and Richards, 2014). The source-sink relationship is one of the most critical physiological determinants of yield, wherein “source” refers to photosynthetically active tissues like leaves and “sink” refers to developing siliquae and seeds that accumulate assimilates (Smith et al., 2018). Under drought stress conditions, weak source strength or limited sink capacity often leads to stagnation in seed yield.

Relatives of *B. juncea* possess tolerance to abiotic stresses such as drought, salinity and heat stress (Malik, 1990; Fletcher et al., 2015; Limbalkar et al., 2021). *Brassica carinata* (Ethiopian mustard), a tetraploid wild relative of *B. juncea*, offers a novel genetic variability for biotic and abiotic stress tolerance traits such as enhanced water-use efficiency, stay-green characteristics, and improved photosynthetic capacity (Getinet et al., 1996; Gaeta et al., 2007; Jiang et al., 2007). The development of *B. carinata*-derived introgression lines (ILs) in *B. juncea* represents a strategic breeding approach to incorporate such traits into elite backgrounds. Several studies have demonstrated the potential of these ILs to perform better under drought, but a comprehensive analysis of their source–sink dynamics under water-deficit conditions has not been fully explored (Wei et al., 2016; Limbalkar et al., 2021; Limbalkar et al., 2023; Vasisth et al., 2023).

In this endeavor, the Division of Genetics, ICAR–Indian Agricultural Research Institute (ICAR-IARI), New Delhi, has developed a set of *B. carinata*-derived *B. juncea* introgression lines (ILs). These ILs incorporate novel genomic regions from *B. carinata* into the *B. juncea* background, thereby generating selectable and exploitable genetic variability (Limbalkar et al., 2024). Previous genetic studies have revealed that these ILs exhibit superior seed yield performance, particularly under drought-stress conditions, validating their use in stress-resilient mustard breeding programs (Limbalkar et al., 2021; Limbalkar et al., 2023). However, the physiological mechanisms underlying source-sink relationships in these ILs under drought conditions remain inadequately explored. A better understanding of these dynamics can unlock further improvements in seed yield by refining selection criteria based on physiological efficiency under moisture deficit stress conditions.

Therefore, the present study was undertaken to characterize *B. carinata*-derived *B. juncea* introgression lines and to establish the source-sink relationship in Indian mustard under moisture-deficit stress conditions. The study aimed to determine whether improvements in seed yield arise from enhanced source strength, increased sink capacity, or a more efficient balance between the two. The findings have provided a comprehensive physiological basis for targeted improvement of mustard cultivars suited to the moisture-deficit stress conditions.

## 2 Materials and methods

### 2.1 Development of introgression lines

A total of 191 ILs carrying genomic segments from *Brassica carinata* in the *B. juncea* genetic background were developed through interspecific hybridization. Three *B. juncea* cultivars, viz., Pusa Agrani, DRMRIJ 31, and Pusa Mustard 30, were crossed with *B. carinata* accession BC 4. The resulting F_2_ populations underwent biparental mating among phenotypically selected plants within each cross to enhance genetic recombination. Stringent phenotypic selection from the F_2_ to F_6_ generations led to the development of three sets of homozygous ILs comprising 78, 87, and 26 in the genetic backgrounds of Pusa Agrani, DRMRIJ 31, and Pusa Mustard 30, respectively).

### 2.2 Evaluation of *Brassica carinata-*derived *B. juncea* introgression lines

A set of 191 ILs, along with their respective parental lines, was evaluated across two locations: ICAR-IARI, New Delhi (L1), and ICAR-DRMR, Bharatpur (L2), under both rainfed and irrigated conditions. The trials were conducted during the *rabi* seasons of 2018–19 and 2020–21. The evaluation of the introgression lines could not be possible during the *rabi* season of 2019–20 due to disruptions caused by the COVID-19 pandemic, which affected field trials. Therefore, the trials were conducted during *rabi* 2018–19 and subsequently repeated in *rabi* 2020–21 to ensure reliable phenotypic evaluation under moisture deficit stress conditions. In 2018–19, the experiment was conducted solely at ICAR-IARI, whereas in 2020–21 it was conducted at both locations, resulting in a total of 6 environments. To assess the variation among ILs for various morpho-physiological traits and their responses to moisture deficit stress, the experiment was laid out in an augmented randomized complete block design with four blocks under both rainfed and irrigated conditions. Standard check varieties, Pusa Agrani, DRMRIJ 31, and Pusa Mustard 30 were included in each block. Each check was replicated twice in each block. Rainfed plots received no supplemental irrigation, while irrigated plots received two irrigations of 50 mm each at 45 and 90 days after sowing (DAS). The row-to-row and plant-to-plant spacing was maintained at 30 cm and 15 cm, respectively. Each plot consisted of a paired row of four meters in length. One row was left unsown before and after each plot, ensuring a plot-to-plot and intra-plot row spacing of 60 cm and 30 cm, respectively, resulting in a total plot area of 2.4 m². Recommended agronomic practices were followed for crop management.

### 2.3 Observations recorded

Observations were recorded on 20 quantitative traits: plant height (cm) at flowering (PH1) and maturity (PH2), number of primary and secondary branches, main shoot length (cm), siliqua length (cm), number of seeds per siliqua, seed yield per plant (g), harvest index, 1,000-seed weight (g), oil content (%), days to 50% flowering, days to maturity, dry matter at flowering (DW1) and maturity (DW2), chlorophyll content at flowering (Chl1) and maturity (Chl2), canopy temperature depression at flowering (CTD1) and maturity (CTD2) and leaf are index at flowering (LAI1) and maturity (LAI2). The harvest index was calculated using dry matter at maturity as the biological yield. Trait data, except days to 50% flowering and days to maturity, were collected from five randomly selected, competitive plants per plot. The latter two traits were recorded on a plot basis. Dry weights were measured at flowering and maturity by drying the harvested plants in a hot air oven. Separate sets of representative plants were randomly sampled within each plot at flowering (DW1) and maturity (DW2). Mean values from multiple sampled plants were used to minimize the influence of micro-environmental variation within the plot. These observations were recorded at the Delhi location during *rabi* 2018–19 (E1) and 2020–21 (E2) seasons. At the Bharatpur location (E3) during the *rabi* season of 2020–21, mostly traits were recorded except for plant height (cm) at flowering (PH1), dry matter at flowering (DW1), chlorophyll content, canopy temperature depression and leaf area index. At the Bharatpur environment (E3) during *rabi* 2020–21, physiological traits could not be recorded due to logistical constraints. However, seed yield and its related traits were recorded, and these data were used for multi-environment evaluation and stability assessment of the introgression lines, while source–sink relationship parameters were estimated only from environments where physiological measurements were available.

Physiological traits, including chlorophyll content (Chl), canopy temperature depression (CTD) and leaf area index (LAI), were recorded at flowering and maturity under both rainfed and irrigated conditions for E1 and E2 environments. Chlorophyll content and CTD were assessed using a handheld SPAD chlorophyll meter (Konica Minolta SPAD-502Plus) and an infrared thermometer (Agri-Therm II), respectively. Trait values were calculated using the following equations:

- Chlorophyll content (nmol/mg fresh weight) = 0.0007 × (SPAD value)² + 0.023 × SPAD value + 0.0544 (Ling et al., 2011)
- Canopy temperature depression (CTD) = Air temperature (Ta) − Canopy temperature (Tc)

Leaf area and leaf area index (LAI) were measured using five randomly selected plants per plot and calculated as follows:

- Leaf Area (LA) = Length (cm) × Width (cm) × 0.6 (Lal and Rao, 1951)
- Leaf Area Index (LAI) = (Leaf Area (cm²) × Number of leaves per plant)/Ground area (cm²) (Blanco and Folegatti, 2003).

Crop growth rate and net assimilation rate were measured using the following formulae suggested by Watson (1952) and Radford (1967);


$$\text{Crop growth rate}(\text{CGR};\text{ g}/\text{day}/{\text{m}}^{2})=\frac{DW_{2}-DW1}{t_{2}-t_{1}}\times \frac{1}{A}$$

$$\text{Net assimilation rate}(\text{NAR};\text{ g}/\text{day}/{\text{m}}^{2})=\frac{DW_{2}-DW1}{t_{2}-t_{1}}\times \frac{\text{ln}L2-\text{ln}L1}{L2-L1}$$

Where, DW_1_ and DW_2_= dry matter accumulation at flowering (t_1_) and maturity (t_2_); A= ground area per plant (0.15 × 0.30 m^2^); ln= natural logarithm; L_1_ and L_2_ = leaf area (m^2^) at t_1_ and t_2_ respectively.

### 2.4 Estimation of source–sink ratio

Dry matter accumulation (DMA) was recorded at two critical growth stages: flowering or pre-reproductive (DW1) and maturity or post-reproductive (DW2). The increment in biomass during the reproductive phase was calculated as:


DMAR=(DW2−DW1)−SY

Where, DMAR = dry matter accumulation at the reproductive phase (excluding seed yield) and SY = seed yield per plant.

The source–sink ratio (SSR) was computed as:

SSR=DMAR/SYIn this context, DW1 represents the pre-reproductive biomass (source potential prior to seed filling), while DW2 represents the total above-ground biomass at maturity. The difference between these two stages reflects the net biomass accumulated during the reproductive period.

The source–sink relationship refers to the balance between assimilate production by photosynthetic organs (source) and their utilization by developing organs such as seeds (sink). Seed yield in crops is largely determined by the coordination between source activity and sink strength, which together regulate assimilate partitioning during reproductive growth (Paul and Foyer, 2001; Reynolds et al., 2005; Korner, 2015).

This ratio provided an estimate of the balance between assimilate supply (source) and its utilization in reproductive sinks. This approach is commonly used in crop physiological studies to quantify source–sink balance and its influence on yield formation in seed crops (Brun and Betts, 1984; Smith et al., 2018; Ibrahim et al., 2021).

### 2.5 Estimation of partitioning efficiency

Partitioning efficiency (PE) was calculated as the proportion of assimilates partitioned into economic yield relative to total reproductive dry matter, using the following formula:

PE=SY/DMARThis index reflects the efficiency with which assimilates accumulated during the reproductive phase were partitioned towards seed yield (Donald and Hamblin, 1976).

### 2.6 Statistical and genetic analyses

Phenotypic data for agro-morphological and physiological traits were analyzed using a mixed linear model incorporating the effects of treatments, checks, genotypes, blocks, and genotype × check interactions. The analysis followed an augmented randomized complete block design (RCBD) as described by Federer (1961). The statistical model was expressed as:


$$Y_{ijk}=\mu +g_{i}+b_{k}+e_{ilk}$$

Where, *Yijk* ​ represents the mean performance of the *ith and jth* genotype over the *kth* block; μ is the overall population mean; *gi*​ is the effect of the *ith* genotype; *b_k_​* denotes the block (replication) effect; and *e_ilk_* ​ is the residual error.

For multi-environment analysis, environments were treated as random effects, while genotypes were considered fixed effects. Genotype × environment (G × E) interactions were estimated using combined analysis across environments based on BLUP values, allowing assessment of genotype stability and performance under contrasting moisture regimes. The significance and magnitude of G × E interactions were interpreted to understand differential genotype responses to environmental conditions, particularly under rainfed and irrigated treatments.

Data analysis was performed using the R package “augmentedRCBD” (version 0.1.5; Aravind et al., 2021) implemented in R version 4.5.1 (version 4.5.1; R Core Team, 2025). Best linear unbiased predictors (BLUPs) were estimated for combined analyses across environments under both rainfed and irrigated conditions using the augmented complete block design module in ACBD-R software (Rodriguez et al., 2018; Lozada and Carter, 2019).

Box–violin plots were generated using Jamovi software (version 2.6; The jamovi project, 2024) to visualize the distribution and variability of agro-morphological traits among ILs under irrigated and rainfed conditions. BLUP values across environments for all traits were subsequently used to estimate Pearson’s correlation coefficients (r) using the “metan” R package (version 1.16.0; Olivoto and Lucio, 2020). Path coefficient analysis was conducted using the R package “agricolae” (de Mendiburu, 2021). Scatter plots were generated using “ggplot2” R package (Wickham, 2016a; 2016b) to illustrate the relationship between seed yield and partitioning efficiency as well as seed yield and source–sink ratio. For visualization, genotypes were color-coded to highlight top-performing ILs in comparison with parental checks.

To examine the relationships among ILs for seed yield and physiological traits under rainfed conditions, a principal component analysis (PCA) was performed using BLUP estimates for all traits. The traits such as seed yield per plant (SY/pt), source–sink ratio, partition efficiency (PE), dry matter accumulation at the reproductive stage (excluding SY), crop growth rate (CGR), net assimilation rate (NAR), leaf area indices (LAI1 and LAI2), canopy temperature depression (CTD), chlorophyll content, and flowering duration were used for PCA analysis. The analysis was performed using R software (version 4.5.1; R Core Team, 2025) with the help of the ‘factoextra’ package (version 1.0.7; Kassambara and Mundt, 2017). The first two principal components were extracted and used to construct the biplot, which was then used to visualize IL–trait associations, identify trait-specific superior genotypes, and detect ILs with stable performance across multiple traits under rainfed conditions.

## 3 Results

The morphological characteristics of the ILs used in this study have been comprehensively established in our earlier study (Limbalkar et al., 2024), which reported high phenotypic uniformity and close resemblance of ILs to their respective *B. juncea* recipient parents. Further, meiotic analyses confirmed normal chromosomal behavior, with all ILs displaying 18 bivalents, identical to the chromosome number of *B. juncea*. The lines also exhibited >99% pollen fertility under both irrigated and rainfed environments, confirming cytological stability and reproductive normalcy. However, the present study utilized these ILs for physiological and agronomic evaluations under moisture deficit stress conditions. Further, it primarily focuses on assessing their drought-response traits, source–sink dynamics, and yield-associated physiological parameters across contrasting moisture environments.

### 3.1 Analysis of variance for morphophysiological traits

In our earlier study, significant variability was observed among ILs for most agromorphological traits (Limbalkar et al., 2024). In addition, the present study further evaluated a range of physiological parameters and yield-related traits. The analysis of variance revealed highly significant differences among the ILs for all the agro-morphological traits recorded across environments (**Table 1**; **Supplementary Tables**), indicating sufficient genetic variability within the ILs for morphophysiological traits. Significant differences were observed for the mean sum of squares between ILs and their parents (test vs. check) for traits, viz., plant height at flowering and maturity, seed yield per plant, days to 50% flowering, and days to maturity in both E1 and E2 under rainfed conditions (**Table 1**). ILs exhibited significant genetic variation for all traits in the present study across environments under both rainfed and irrigated conditions. Under irrigated conditions, the analysis of variance indicated significant differences among ILs for all traits in E1 and E2 environments. The ILs vs. check comparisons were also significant for most of the studied traits across environments under irrigated conditions (**Table 1**).

**Table 1:** Analysis of variance of introgression lines (ILs) and parents for studying different agro-morphological traits under rainfed (RF) and irrigated (IR) conditions.

Source of variations		Block		Treatment		ILs		Checks		ILs vs. checks		Residuals		CV (%)	
*Df*		3		194		191		2		1		18			
Traits	Conditions	E1	E2	E1	E2	E1	E2	E1	E2	E1	E2	E1	E2	E1	E2
PHF	RF	1941.89**	62.86ns	530.25**	576.61**	519.34**	537.83**	716.42**	633.46**	2241.83**	7869.73**	41.65	100.36	7.53	9.84
	IR	27.05ns	1057.38**	683.7**	557.7**	675.61**	506.6**	146.41ns	628.96*	3302.18**	10175.95**	50.18	152.16	7.09	13.7
PHM	RF	395.42**	251ns	322.82**	279.72*	298.59**	258.99*	1726.81**	918.36**	2143.57**	2961.54**	41.39	126.13	3.22	5.57
	IR	80.09*	426.55**	430.44**	303.68**	379.73**	272.17**	1235.65**	845.09**	8505.8**	5238.24**	24.93	60.02	2.34	3.68
DMF	RF	18.96ns	0.06ns	246.52**	58.23**	250.16**	57.5**	21.74ns	50.6**	0.06ns	213.41**	18.73	1.69	10.51	5.26
	IR	73.78ns	0.01ns	201.27**	67.65**	196.45**	61.5**	6.17ns	11.18*	1511.73**	1354.35**	42.59	1.98	13.65	5.57
DMM	RF	609.94**	114.5ns	1072.83**	465.66**	990.58**	436.24**	6437.17**	3503.41**	6052.97**	9.14ns	72.54	46.1	7.61	7.11
	IR	537.05**	53.08ns	2026.44**	548.62**	1840.73**	531.47**	417.17**	2150.75**	40716.26**	619.68**	43.87	46.4	5.67	6.58
SY/pt	RF	24.07*	7.5ns	76.8**	36.2**	74.87**	34.38**	215.81**	206.31**	168.7**	42.25**	6.48	4.06	11.41	8.57
	IR	25.96**	7.01ns	71.3**	43.06**	69.28**	42.38**	23.18**	129.38**	552.57**	0.58ns	1.99	8.95	6.9	12.26
D50%F	RF	8.22ns	0.22ns	67.15**	21.65**	60.18**	19.9**	244.5**	126.17**	1043.47**	145.84**	4.69	0.57	4.03	1.54
	IR	36.12**	1.06ns	66.98**	31.08**	60.74**	28.29**	3.5ns	100.5**	1386.75**	425.03**	1.42	0.91	2.12	1.88
DM	RF	5.78**	4.17*	14.78**	9.36**	12.9**	8.3**	193.17**	98.17**	17.93**	34.17**	0.31	1.17	3.36	4.76
	IR	24.38**	0.89ns	22.2**	4.83**	20.19**	4.3**	6ns	45.5**	440.04**	24.32**	1.91	0.57	2.91	3.52

*df*, degrees of freedom; E1 and E2 are evaluation during *rabi* season 2018–19 and 2020–21 season respectively; PH45, Plant height (cm) at flowering; PH90, Plant height (cm) at maturity; DMF, Dry matter at flowering (g); DMM, Dry matter at maturity (g); SY/pt, Seed yield per plant (g); SY/plot, Seed yield per plot (g); D50%F, Days to 50% flowering; DM, Days to maturity **Significant at *P* = 0.01 and *significant at *P* = 0.05, ns= non–significant. The residual error was estimated from the replicated checks, and the residual degrees of freedom were derived from the block × check interaction and within-check replication components.

### 3.2 Variability among introgression lines for morpho−physiological traits

**Figure 1:**
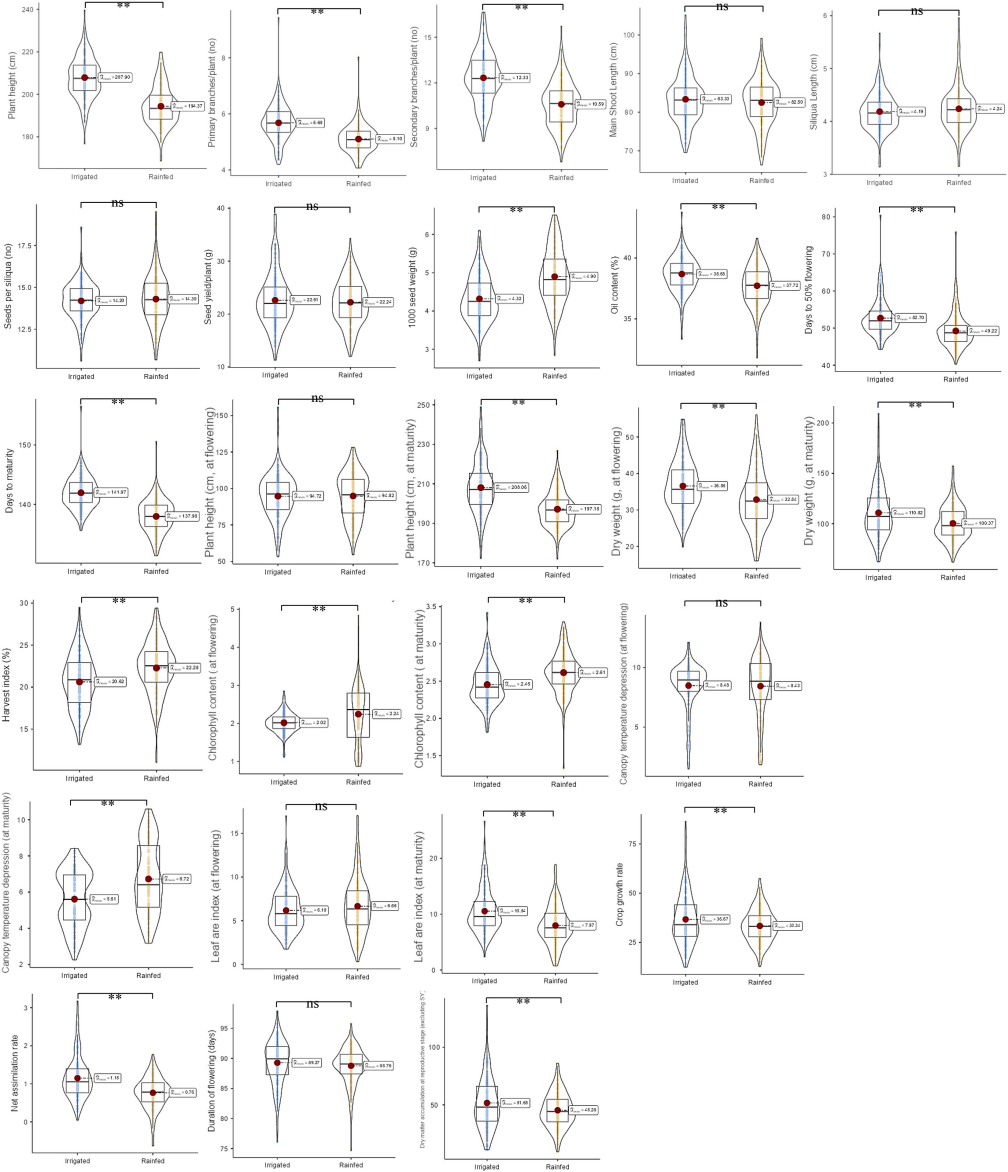
Violin plots overlaid with box plots for agro-morphological traits in introgression lines (ILs) under irrigated and rainfed conditions across environments. Where, **p < 0.01; ns, non-significant.

Sufficient phenotypic variation was observed for agro-morphological and physiological traits among ILs evaluated under rainfed and irrigated conditions across environments. Most of the traits exhibited a normal distribution (**Figure 1**). Significant differences were observed in mean performance between rainfed and irrigated conditions for morpho-physiological traits, including plant height at maturity (PH2), primary and secondary branches per plant, 1,000 seed weight, oil content, days to 50% flowering, days to maturity, dry weight at flowering (DW1) and maturity (DW2), harvest index, chlorophyll content at flowering (Chl1) and maturity (Chl2), canopy temperature depression at maturity (CTD2), leaf area index at maturity (LAI2), crop growth rate (CGR), net assimilation rate (NAR) and dry matter accumulation at reproductive stage (excluding seed yield). Mean values of 1,000-seed weight, harvest index, Chl1, Chl2, and CTD2 were found to be significantly higher under rainfed conditions than under irrigated conditions (**Figure 1**). In contrast, no significant differences between rainfed and irrigated conditions were observed for main shoot length, siliqua length, seeds per siliqua, seed yield per plant, plant height at flowering (PH1), CTD1, LAI1, and duration of flowering. Whereas the remaining traits exhibited significantly higher mean values under irrigated conditions compared with rainfed conditions (**Figure 1**).

### 3.3 Association among agro-morphological and physiological traits

**Figure 2:**
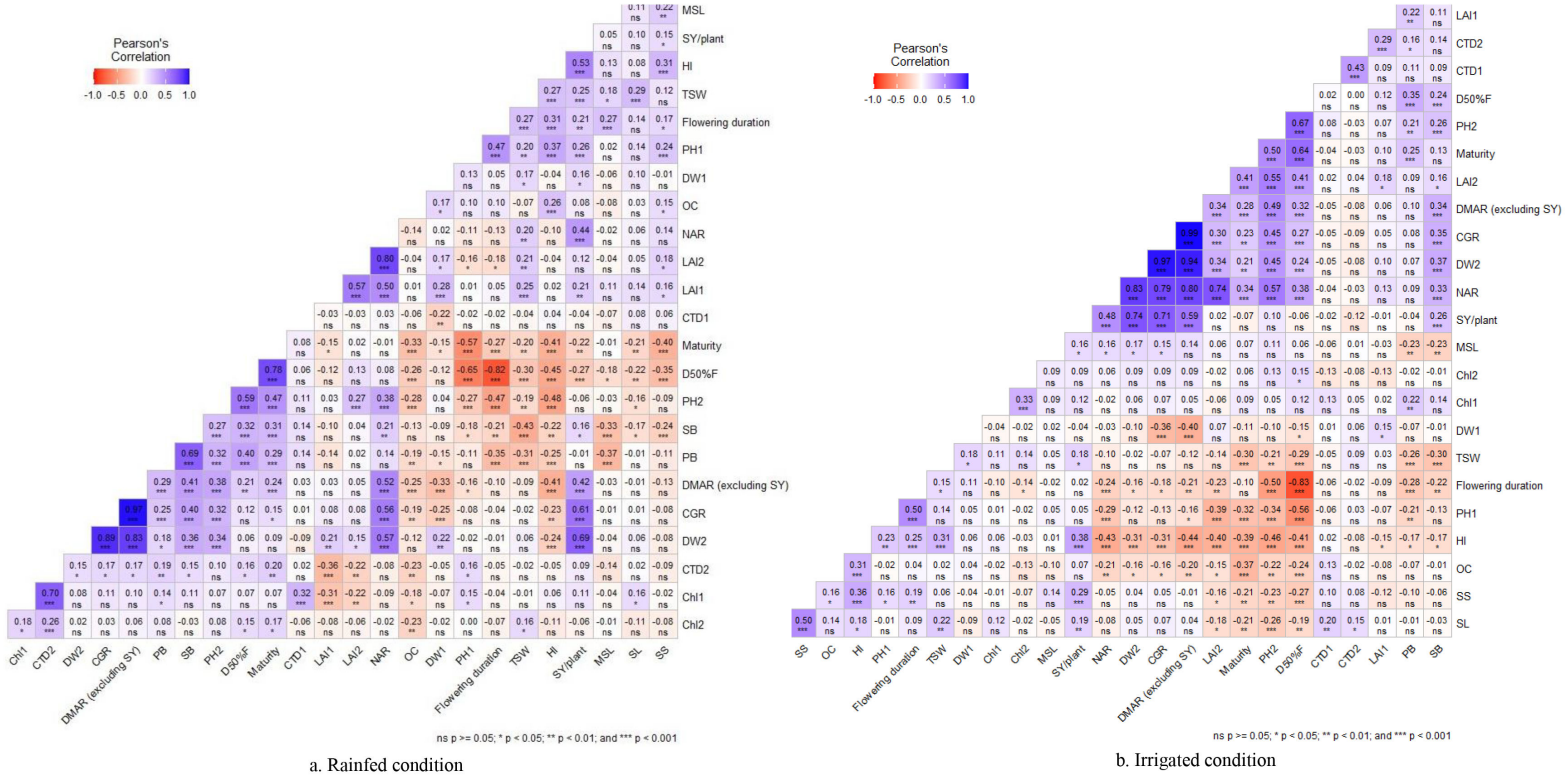
Correlation coefficients between seed yield contributing as well as physiological traits under rainfed **(A)** and irrigated **(B)** conditions across environments. PH1 & PH2, Plant height at flowering and maturity, respectively (g); PB, primary branches per plant, SB, secondary branches per plant, MSL, main shoot length (cm), SL, siliqua length (cm); SS, seeds per siliqua; TSW ,  1,000 seed weight (g); SY/pt, seed yield per plant (g); HI, harvest index (%); OC, oil content (%); D50%F, days to 50% flowering; Maturity, days to maturity; DW1& DW2, dry matter accumulation at flowering and maturity, respectively (g); Chl1&Chl2, chlorophyll content at flowering and maturity, respectively; CTD1 &CTD2, canopy temperature depression at flowering and maturity, respectively (°C); LAI 1&2, leaf area index at flowering and maturity, respectively; CGR, crop growth rate; NAR, net assimilation rate; DMAR, dry matter accumulation at reproductive stage (g).

Seed yield per plant (g), a major sink trait, is influenced by the coordinated interaction of several source- and sink-related agro-morphological and physiological traits. In the present study, Pearson’s correlation analysis was performed to elucidate the source–sink relationships under rainfed and irrigated conditions (**Figures 2A, B**). Results revealed that seed yield per plant (g) showed a significant positive correlation (*P* ≤ 0.01) with traits such as dry matter accumulation at maturity (g; r = 0.69), crop growth rate (r = 0.61), dry matter accumulation (excluding seed yield; r = 0.42), leaf area index (r = 0.21), net assimilation rate (r = 0.44), plant height at flowering (r = 0.26), flowering duration (r = 0.21), 1,000 seed weight (r = 0.25) and harvest index (r = 0.53), whereas, negatively associated with days to 50% flowering (r = − 0.31) and days to maturity (r = − 0.31) under moisture deficit stress conditions (**Figure 2A**). Under irrigated conditions, seed yield per plant exhibited a positive and significant correlation (*P* ≤ 0.01) with net assimilation rate (r = 0.48), dry matter accumulation at maturity (g; r = 0.74), crop growth rate (r = 0.71), dry matter accumulation (excluding seed yield; r = 0.59), siliqua length (r = 0.19), seeds per siliqua (r = 0.29), harvest index (r = 0.38) and number of secondary branches per plant (r = 0.26), whereas, negatively associated with days to 50% flowering (r = − 0.31) and days to maturity (r = − 0.31).

Furthermore, under rainfed conditions, dry matter accumulation at the reproductive stage (excluding seed yield) exhibited strong and significant positive correlations (P < 0.001) with dry matter accumulation at maturity, crop growth rate, primary and secondary branches per plant, plant height at maturity, days to 50% flowering, days to maturity, net assimilation rate and seed yield per plant (**Figure 2A**).

Net assimilation rate showed a significant and positive association with dry matter accumulation at maturity, crop growth rate, dry matter accumulation at the reproductive stage (excluding seed yield), plant height at maturity, leaf area index at flowering and maturity, 1,000 seed weight and seed yield per plant under rainfed conditions. Apart from seed yield, 1,000-seed weight is an important trait that contributes to it. In the present study, a significant positive correlation was observed between 1,000 seed weight and leaf area index at flowering and maturity, net assimilation rate, flowering duration, harvest index, seed yield per plant and siliqua length under moisture deficit stress conditions.

Path coefficient analysis (**Table 2**) revealed that, under moisture deficit stress conditions, dry matter accumulation at maturity (g; ρ = 1.08) followed by harvest index (%; ρ = 0.58), crop growth rate (ρ = 0.42) and net assimilation rate (ρ = 0.09) had a higher positive direct effect on the expression of seed yield per plant (g). Further, higher positive indirect effects of dry matter accumulation (excluding seed yield; ρ = 1.15), net assimilation rate (ρ = 0.35), dry matter accumulation at flowering (ρ = 0.34), 1,000 seed weight (ρ = 0.26), plant height at flowering (ρ = 0.22), flowering duration (ρ = 0.22), leaf area index at flowering (ρ = 0.21), crop growth rate (ρ = 0.19), secondary branches per plant (ρ = 0.18), seeds per siliqua (ρ = 0.15), siliqua length (ρ = 0.11), chlorophyll content at flowering (ρ = 0.11) and canopy temperature depression at maturity (ρ = 0.11) were observed on seed yield per plant (g). Under moisture-deficit stress conditions, negative direct effects on seed yield per plant were observed for dry matter accumulation excluding seed yield (ρ = −0.73) and dry matter accumulation at flowering (ρ = −0.18). In contrast, dry matter accumulation at maturity, days to 50% flowering, and days to maturity exhibited higher negative indirect effects on seed yield per plant under rainfed conditions (**Table 2**). Similarly, under irrigated conditions (**Table 3**), dry matter accumulation at maturity (ρ = 2.04), days to 50% flowering (ρ = 1.82), flowering duration (ρ = 1.41), crop growth rate (ρ = 0.95), harvest index (ρ = 0.28) and net assimilation rate (ρ = 0.10) were observed to have higher positive direct effects on seed yield per plant (g). The positive indirect effects on seed yield per plant were observed through traits like dry matter accumulation excluding seed yield (ρ = 2.96), days to maturity (ρ = 0.93), dry matter accumulation at flowering (ρ = 0.41), net assimilation rate (ρ = 0.38), seed per siliqua (ρ = 0.27), secondary branches (ρ = 0.24), siliqua length (ρ = 0.19), main shoot length (ρ = 0.17), 1,000 seed weight (ρ = 0.17), harvest index (ρ = 0.10), chlorophyll content (ρ = 0.10) and leaf area index at maturity (ρ = 0.10).

**Table 2:** A path coefficient analysis showing total, direct, and indirect effects of seed yield contributing traits and physiological traits on seed yield (SY) per plant in ILs under rainfed condition across environments.

Traits	PH1	PH2	PB	SB	MSL	SL	SS	TSW	OC	D50F	Maturity	DW1	DW2	HI	Chl1	Chl2	CTD1	CTD2	LAI1	LAI2	CGR	NAR	FD	DMAR	Direct	Indirect	Correlation with SY
PH1		0.01	0.00	0.00	0.00	0.00	0.00	0.00	0.00	-0.01	-0.02	-0.02	-0.02	0.21	0.00	0.00	0.00	0.00	0.00	0.01	-0.03	-0.01	0.00	0.12	0.04	0.22	0.26**
PH2	-0.01		0.01	0.00	0.00	0.00	0.00	0.00	0.00	0.01	0.01	-0.01	0.37	-0.28	0.00	0.00	0.00	0.00	0.00	-0.01	0.13	0.03	0.00	-0.28	-0.04	-0.02	-0.06
PB	0.00	-0.01		-0.01	0.00	0.00	0.00	0.00	0.00	0.01	0.01	0.03	0.19	-0.14	0.00	0.00	0.00	0.00	0.00	0.00	0.10	0.01	0.00	-0.21	0.02	-0.03	-0.01
SB	-0.01	-0.01	0.01		0.00	0.00	0.00	0.00	0.00	0.01	0.01	0.02	0.39	-0.13	0.00	0.00	0.00	0.00	0.00	0.00	0.17	0.02	0.00	-0.30	-0.02	0.18	0.16*
MSL	0.00	0.00	-0.01	0.01		0.00	0.00	0.00	0.00	0.00	0.00	0.01	-0.04	0.08	0.00	0.00	0.00	0.00	0.00	0.00	0.00	0.00	0.00	0.02	-0.01	0.06	0.05
SL	0.00	0.01	0.00	0.00	0.00		0.00	0.00	0.00	0.00	-0.01	-0.02	0.06	0.05	0.00	0.00	0.00	0.00	0.00	0.00	0.00	0.01	0.00	0.01	-0.01	0.11	0.10
SS	0.01	0.00	0.00	0.00	0.00	0.00		0.00	0.00	-0.01	-0.01	0.00	-0.09	0.18	0.00	0.00	0.00	0.00	0.00	-0.01	-0.03	0.01	0.00	0.10	0.00	0.15	0.15*
TSW	0.01	0.01	-0.01	0.01	0.00	0.00	0.00		0.00	-0.01	-0.01	-0.03	0.06	0.16	0.00	0.00	0.00	0.00	0.00	-0.01	-0.01	0.02	0.00	0.07	-0.01	0.26	0.25**
OC	0.00	0.01	0.00	0.00	0.00	0.00	0.00	0.00		0.00	-0.01	-0.03	-0.13	0.15	0.00	0.00	0.00	0.00	0.00	0.00	-0.08	-0.01	0.00	0.18	-0.01	0.09	0.08
D50%F	-0.02	-0.03	0.01	-0.01	0.00	0.00	0.00	0.00	0.00		0.02	0.02	0.06	-0.26	0.00	0.00	0.00	0.00	0.00	-0.01	0.05	0.01	0.01	-0.15	0.02	-0.29	-0.27
Maturity	-0.02	-0.02	0.00	-0.01	0.00	0.00	0.00	0.00	0.00	0.01		0.03	0.10	-0.24	0.00	0.00	0.00	0.00	0.00	0.00	0.06	0.00	0.00	-0.18	0.03	-0.25	-0.22
DW1	0.00	0.00	0.00	0.00	0.00	0.00	0.00	0.00	0.00	0.00	0.00		0.24	-0.02	0.00	0.00	0.00	0.00	0.00	-0.01	-0.10	0.00	0.00	0.24	-0.18	0.34	0.16*
DW2	0.00	-0.01	0.00	-0.01	0.00	0.00	0.00	0.00	0.00	0.00	0.00	-0.04		-0.14	0.00	0.00	0.00	0.00	0.00	-0.01	0.37	0.05	0.00	-0.61	1.08**	-0.39	0.69**
HI	0.01	0.02	0.00	0.00	0.00	0.00	0.00	0.00	0.00	-0.01	-0.01	0.01	-0.26		0.00	0.00	0.00	0.00	0.00	0.00	-0.10	-0.01	0.00	0.30	0.58**	-0.05	0.53**
Chl1	0.01	0.00	0.00	0.00	0.00	0.00	0.00	0.00	0.00	0.00	0.00	0.01	0.09	0.03		0.00	0.00	-0.01	0.00	0.01	0.05	-0.01	0.00	-0.07	0.00	0.11	0.11
Chl2	0.00	0.00	0.00	0.00	0.00	0.00	0.00	0.00	0.00	0.00	0.00	0.00	0.02	-0.06	0.00		0.00	-0.01	0.00	0.00	0.01	0.00	0.00	-0.04	0.01	-0.07	-0.06
CTD1	0.00	0.00	0.00	0.00	0.00	0.00	0.00	0.00	0.00	0.00	0.00	0.04	-0.10	0.02	0.00	0.00		0.00	0.00	0.00	0.00	0.00	0.00	-0.02	0.01	-0.05	-0.04
CTD2	0.01	0.00	0.00	0.00	0.00	0.00	0.00	0.00	0.00	0.00	0.01	0.01	0.16	-0.03	0.00	0.00	0.00		0.00	0.01	0.07	-0.01	0.00	-0.12	-0.02	0.11	0.09
LAI1	0.00	0.00	0.00	0.00	0.00	0.00	0.00	0.00	0.00	0.00	0.00	-0.05	0.23	0.01	0.00	0.00	0.00	0.01		-0.02	0.03	0.04	0.00	-0.02	0.00	0.21	0.21**
LAI2	-0.01	-0.01	0.00	0.00	0.00	0.00	0.00	0.00	0.00	0.00	0.00	-0.03	0.16	-0.02	0.00	0.00	0.00	0.00	0.00		0.03	0.07	0.00	-0.04	-0.04	0.16	0.12
CGR	0.00	-0.01	0.00	-0.01	0.00	0.00	0.00	0.00	0.00	0.00	0.00	0.04	0.96	-0.13	0.00	0.00	0.00	0.00	0.00	0.00		0.05	0.00	-0.71	0.42**	0.19	0.61**
NAR	0.00	-0.02	0.00	0.00	0.00	0.00	0.00	0.00	0.00	0.00	0.00	0.00	0.62	-0.06	0.00	0.00	0.00	0.00	0.00	-0.03	0.23		0.00	-0.38	0.09**	0.35	0.44**
FD	0.02	0.02	-0.01	0.00	0.00	0.00	0.00	0.00	0.00	-0.01	-0.01	-0.01	-0.01	0.18	0.00	0.00	0.00	0.00	0.00	0.01	-0.02	-0.01		0.07	-0.01	0.22	0.21**
DMAR (excl SY)	-0.01	-0.02	0.00	-0.01	0.00	0.00	0.00	0.00	0.00	0.00	0.01	0.06	0.90	-0.24	0.00	0.00	0.00	0.00	0.00	0.00	0.41	0.04	0.00		-0.73	1.15**	0.42**

PH1 & PH2, Plant height at flowering and maturity, respectively (g); PB, primary branches per plant, SB, secondary branches per plant, MSL, main shoot length (cm), SL, siliqua length (cm); SS, seeds per siliqua; TSW ,  1,000 seed weight (g); SY/pt, seed yield per plant (g); HI, harvest index (%); OC, oil content (%); D50%F, days to 50% flowering; Maturity, days to maturity; DW1& DW2, dry matter accumulation at flowering and maturity, respectively (g); Chl1&Chl2, chlorophyll content at flowering and maturity, respectively; CTD1 &CTD2, canopy temperature depression at flowering and maturity, respectively (°C); LAI 1&2, leaf area index at flowering and maturity, respectively; CGR, crop growth rate; NAR, net assimilation rate; DMAR, dry matter accumulation at reproductive stage (g).

* P ≤ 0.05, ** P ≤ 0.01.

**Table 3:** A path coefficient analysis showing total, direct, and indirect effects of seed yield contributing traits and physiological traits on seed yield per plant in ILs under irrigated condition across environments.

Traits	PH1	PH2	PB	SB	MSL	SL	SS	TSW	OC	D50F	Maturity	DW1	DW2	HI	Chl1	Chl2	CTD1	CTD2	LAI1	LAI2	CGR	NAR	FD	DMAR	Direct	Indirect	Correlation with SY
PH1		0.01	0.00	0.00	0.00	0.00	0.00	0.00	0.00	-1.02	0.32	-0.02	-0.24	0.06	0.00	0.00	0.00	0.00	0.00	0.03	-0.12	-0.03	0.70	0.38	-0.02	0.07	0.05
PH2	0.01		0.00	0.00	0.00	0.00	0.00	0.00	0.00	1.22	-0.50	0.04	0.92	-0.13	0.00	0.00	0.00	0.00	0.00	-0.04	0.43	0.06	-0.70	-1.16	-0.03	0.13	0.10
PB	0.00	-0.01		0.01	0.00	0.00	0.00	0.00	0.00	0.64	-0.25	0.03	0.14	-0.05	0.00	0.00	0.00	0.00	0.00	-0.01	0.08	0.01	-0.39	-0.24	-0.01	-0.03	-0.04
SB	0.00	-0.01	0.00		0.00	0.00	0.00	0.00	0.00	0.44	-0.13	0.00	0.75	-0.05	0.00	0.00	0.00	0.00	0.00	-0.01	0.33	0.03	-0.31	-0.81	0.02	0.24	0.26**
MSL	0.00	0.00	0.00	0.00		0.00	0.00	0.00	0.00	0.11	-0.07	-0.01	0.35	0.00	0.00	0.00	0.00	0.00	0.00	0.00	0.14	0.02	-0.03	-0.33	-0.01	0.17	0.16
SL	0.00	0.01	0.00	0.00	0.00		0.01	0.00	0.00	-0.35	0.21	0.04	0.10	0.05	0.00	0.00	0.01	0.00	0.00	0.01	0.07	-0.01	0.13	-0.09	0.00	0.19	0.19**
SS	0.00	0.01	0.00	0.00	0.00	0.00		0.00	0.00	-0.49	0.21	0.02	0.08	0.10	0.00	0.00	0.00	0.00	0.00	0.01	0.05	-0.01	0.27	0.02	0.02	0.27	0.29**
TSW	0.00	0.01	0.00	0.00	0.00	0.00	0.00		0.00	-0.53	0.30	-0.08	-0.04	0.09	0.00	0.00	0.00	0.00	0.00	0.01	-0.07	-0.01	0.21	0.28	0.01	0.17	0.18*
OC	0.00	0.01	0.00	0.00	0.00	0.00	0.00	0.00		-0.44	0.37	-0.02	-0.33	0.09	0.00	0.00	0.00	0.00	0.00	0.01	-0.15	-0.02	0.06	0.47	0.01	0.06	0.07
D50%F	0.01	-0.02	0.00	0.00	0.00	0.00	-0.01	0.00	0.00		-0.64	0.07	0.49	-0.12	0.00	0.00	0.00	0.00	0.00	-0.03	0.26	0.04	-1.17	-0.76	1.82	-1.88	-0.06
Maturity	0.01	-0.02	0.00	0.00	0.00	0.00	0.00	0.00	0.00	1.16		0.05	0.43	-0.11	0.00	0.00	0.00	0.00	0.00	-0.03	0.22	0.03	-0.14	-0.66	-1.00	0.93	-0.07
DW1	0.00	0.00	0.00	0.00	0.00	0.00	0.00	0.00	0.00	-0.27	0.11		-0.20	0.02	0.00	0.00	0.00	0.00	0.00	-0.01	-0.34	0.00	0.15	0.95	-0.45	0.41	-0.04
DW2	0.00	-0.01	0.00	0.01	0.00	0.00	0.00	0.00	0.00	0.44	-0.21	0.04		-0.09	0.00	0.00	0.00	0.00	0.00	-0.03	0.92	0.08	-0.23	-2.23	2.04	-1.30	0.74**
HI	-0.01	0.01	0.00	0.00	0.00	0.00	0.01	0.00	0.00	-0.74	0.39	-0.03	-0.63		0.00	0.00	0.00	0.00	0.00	0.03	-0.30	-0.04	0.35	1.04	0.28	0.10	0.38**
Chl1	0.00	0.00	0.00	0.00	0.00	0.00	0.00	0.00	0.00	0.22	-0.09	0.02	0.12	0.02		0.00	0.00	0.00	0.00	0.00	0.07	0.00	-0.14	-0.12	0.02	0.10	0.12
Chl2	0.00	0.00	0.00	0.00	0.00	0.00	0.00	0.00	0.00	0.27	-0.06	0.01	0.18	-0.01	0.01		0.00	0.00	0.00	0.00	0.09	0.01	-0.20	-0.21	0.01	0.08	0.09
CTD1	0.00	0.00	0.00	0.00	0.00	0.00	0.00	0.00	0.00	0.04	0.04	0.00	-0.10	0.01	0.00	0.00		-0.01	0.00	0.00	-0.05	0.00	-0.08	0.12	0.03	-0.05	-0.02
CTD2	0.00	0.00	0.00	0.00	0.00	0.00	0.00	0.00	0.00	0.00	0.03	-0.03	-0.16	-0.02	0.00	0.00	0.01		0.00	0.00	-0.09	0.00	-0.03	0.19	-0.03	-0.09	-0.12
LAI1	0.00	0.00	0.00	0.00	0.00	0.00	0.00	0.00	0.00	0.22	-0.10	-0.07	0.20	-0.04	0.00	0.00	0.00	-0.01		-0.01	0.05	0.01	-0.13	-0.14	0.01	-0.02	-0.01
LAI2	0.01	-0.02	0.00	0.00	0.00	0.00	0.00	0.00	0.00	0.74	-0.41	-0.03	0.69	-0.11	0.00	0.00	0.00	0.00	0.00		0.29	0.07	-0.32	-0.81	-0.08	0.10	0.02
CGR	0.00	-0.01	0.00	0.01	0.00	0.00	0.00	0.00	0.00	0.49	-0.23	0.16	1.98	-0.09	0.00	0.00	0.00	0.00	0.00	-0.02		0.08	-0.25	-2.35	0.95	-0.24	0.71**
NAR	0.01	-0.02	0.00	0.00	0.00	0.00	0.00	0.00	0.00	0.69	-0.34	0.01	1.69	-0.12	0.00	0.00	0.00	0.00	0.00	-0.06	0.75		-0.34	-1.90	0.10	0.38	0.48**
FD	-0.01	0.02	0.00	0.00	0.00	0.00	0.00	0.00	0.00	-1.51	0.10	-0.05	-0.33	0.07	0.00	0.00	0.00	0.00	0.00	0.02	-0.17	-0.02		0.50	1.41	-1.39	0.02
DMAR (excl SY)	0.00	-0.02	0.00	0.01	0.00	0.00	0.00	0.00	0.00	0.58	-0.28	0.18	1.92	-0.12	0.00	0.00	0.00	0.00	0.00	-0.03	0.94	0.08	-0.30		-2.37	2.96	0.59**

PH1 & PH2, Plant height at flowering and maturity, respectively (g); PB, primary branches per plant, SB, secondary branches per plant, MSL, main shoot length (cm), SL, siliqua length (cm); SS, seeds per siliqua; TSW ,  1,000 seed weight (g); SY/pt, seed yield per plant (g); HI, harvest index (%); OC, oil content (%); D50%F, days to 50% flowering; Maturity, days to maturity; DW1& DW2, dry matter accumulation at flowering and maturity, respectively (g); Chl1&Chl2, chlorophyll content at flowering and maturity, respectively; CTD1 &CTD2, canopy temperature depression at flowering and maturity, respectively (°C); LAI 1&2, leaf area index at flowering and maturity, respectively; CGR, crop growth rate; NAR, net assimilation rate; DMAR, dry matter accumulation at reproductive stage (g).

* P ≤ 0.05, ** P ≤ 0.01.

### 3.4 Performance of ILs and parents across environments

**Figure 3:**
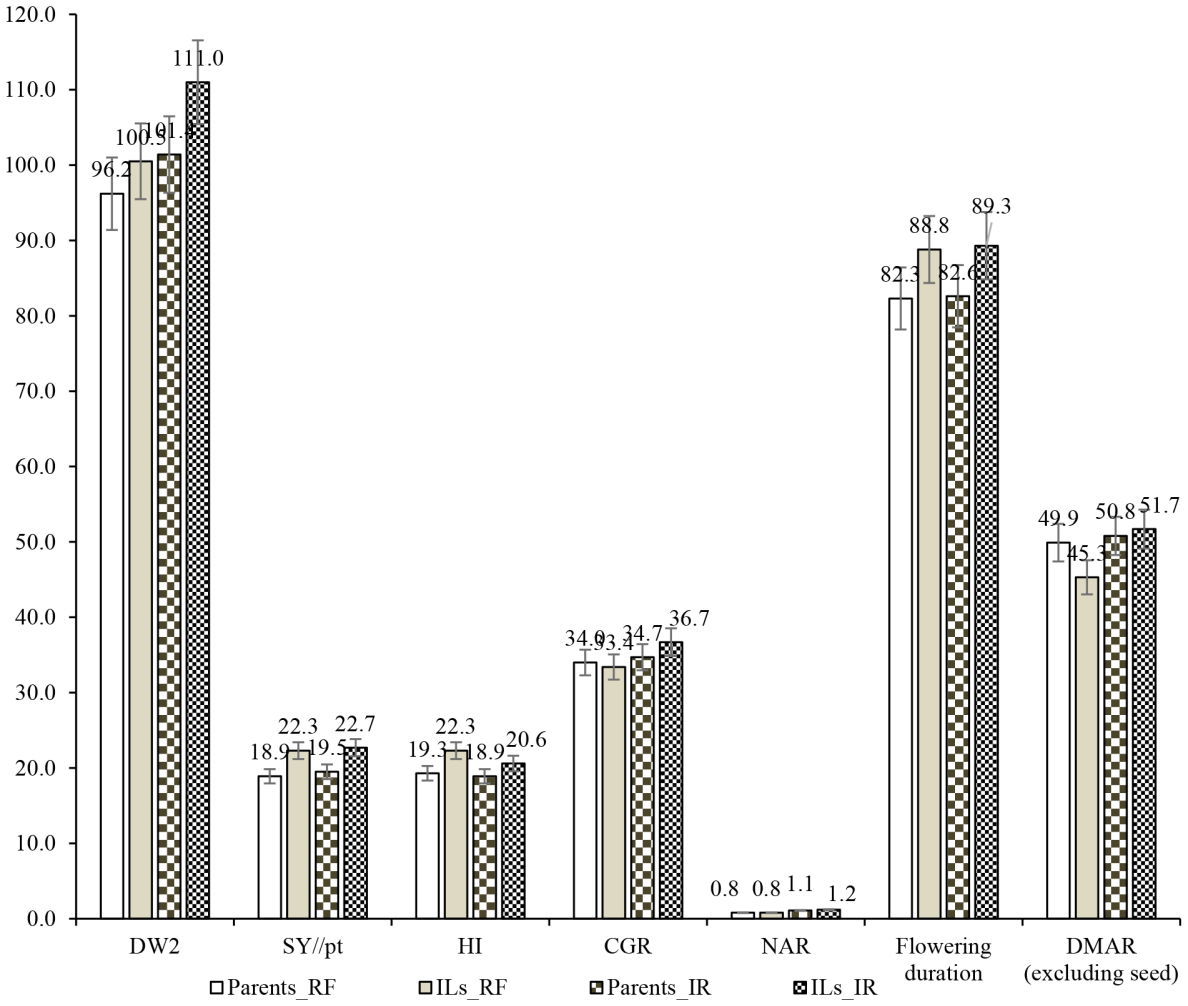
Mean performance of ILs and their parents for different traits under rainfed and irrigated conditions across the environments (DW2 = dry matter accumulation after 90 DAS; SY/pt, seed yield per plant; HI, harvest index; CGR, crop growth rate; NAR, net assimilation rate; DMAR, dry matter accumulation at reproductive stage).

Distinct differences were observed between parents and ILs for growth, yield, and physiological traits associated with water-use efficiency under rainfed and irrigated conditions (**Figure 3**). For dry matter accumulation at maturity (DW2), ILs recorded higher mean values than their parents under both rainfed and irrigated conditions, with the highest DW2 observed in ILs under irrigated conditions.

Seed yield per plant was also higher in ILs than in the parents across both environments, with a marked increase under irrigated conditions. The harvest index (HI) showed higher values in ILs than in their parents under both water regimes. Crop growth rate (CGR) was comparable between ILs and their parents under rainfed conditions, whereas it was higher in ILs than in their parents under irrigated conditions (**Figure 3**). Net assimilation rate remained comparable between parents and ILs under rainfed conditions but increased markedly under irrigated conditions, with ILs recording higher values, suggesting improved assimilatory capacity under a favorable moisture regime.

Flowering duration was longer in ILs than in parents under both rainfed and irrigated conditions. Dry matter accumulation during the reproductive stage showed contrasting responses between environments. Under rainfed conditions, parents recorded slightly higher reproductive dry matter accumulation, whereas under irrigated conditions, ILs accumulated more (**Figure 3**), indicating environment-specific variation in source–sink dynamics. Overall, ILs demonstrated superior performance over parents for DW2, SY/pt, CGR, flowering duration, and DMAR across moisture regimes (**Figure 3**).

### 3.5 Relationship between source–sink traits and seed yield

**Figure 4:**
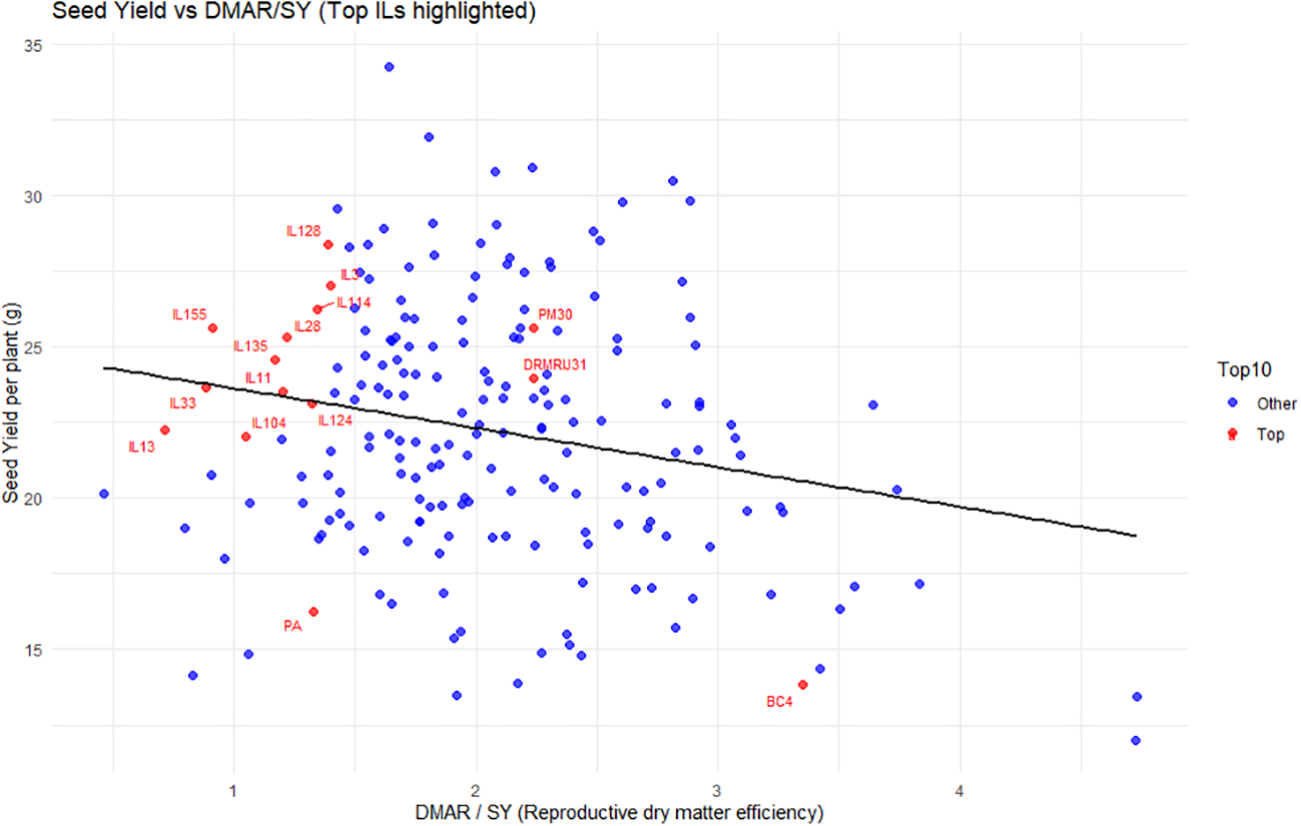
Scatterplot depicting the relationship between source–sink ratio (SSR) and seed yield per plant under moisture deficit stress conditions. Each point represents an introgression line, illustrating variation in source–sink balance and its association with seed yield performance under moisture deficit stress.

The scatterplot illustrates the relationship between reproductive dry matter efficiency, expressed as the source-sink ratio, and seed yield per plant among the introgression lines. A wide range of variation was observed for both source-sink ratio and seed yield per plant, indicating substantial diversity among ILs for source–sink ratio (**Figure 4**).

Most ILs clustered within a moderate range of source-sink ratios, with varying levels of seed yield per plant. Several ILs exhibited relatively higher seed yield per plant at lower to intermediate source-sink ratios. In contrast, lines with higher source-sink ratio values generally showed lower seed yield per plant. The fitted regression line indicated an overall negative trend between the source sink ratio and seed yield per plant in ILs under moisture deficit stress conditions.

Few ILs, viz., IL3, IL11, IL13, IL28, IL33, IL104, IL114, IL124, IL128, IL135, and IL155, positioned close to or above the general trend line, exhibited higher seed yield per plant and source-sink ratio, highlighting superior performance under moisture-deficit stress conditions. Overall, the distribution pattern highlights substantial genotypic variation in the source-sink ratio and its association with seed yield among the ILs.

**Figure 5:**
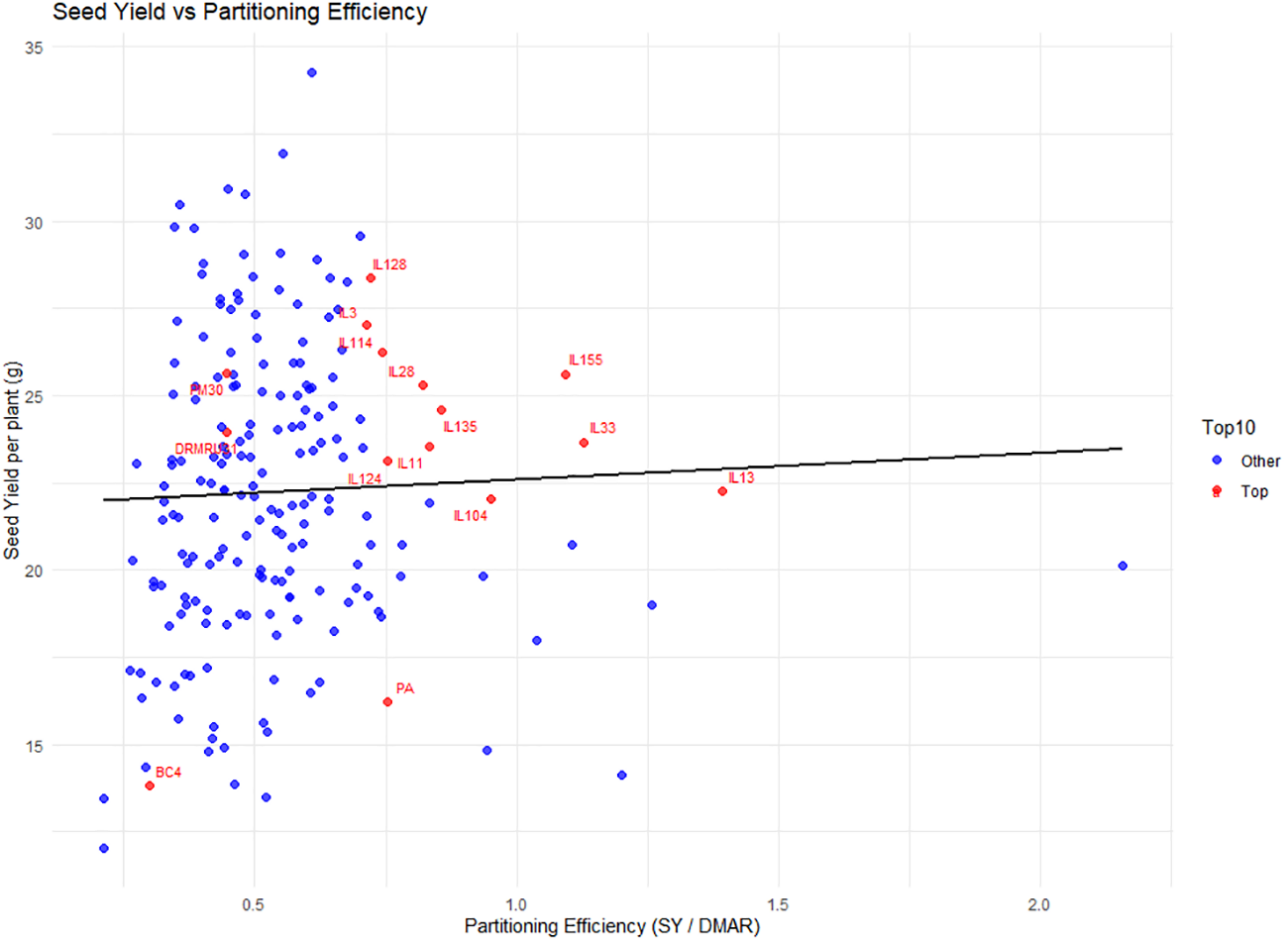
Scatterplot showing the relationship between dry matter partitioning efficiency (PE) and seed yield per plant under moisture deficit stress conditions. The distribution of introgression lines highlights differences in assimilate allocation to reproductive sinks and its influence on yield under moisture deficit stress.

Further, the relationship between dry matter partitioning efficiency and seed yield per plant under rainfed conditions showed wide variation among the introgression lines (**Figure 5**). Most ILs were distributed within a low to intermediate range of partitioning efficiency, with substantial variability in seed yield per plant. The fitted regression line indicated a positive trend between partitioning efficiency and seed yield per plant (**Figure 5**).

Several ILs, including IL11, IL33, IL124, IL128, IL135 and IL155 were positioned above the regression line and recorded comparatively higher seed yield per plant at moderate partitioning efficiency levels, indicating superior performance under rainfed conditions. A few ILs, such as IL3, IL114 and IL28, also exhibited relatively higher seed yield per plant within the main cluster of ILs. In contrast, some genotypes with higher partitioning efficiency values showed comparatively lower seed yield per plant. The parental lines viz., Pusa Agrani, DRMRIJ31, Pusa Mustard 30 and BC4 were scattered across the range of partitioning efficiency with variable yield performance (**Figure 5**). Results also demonstrate considerable genotypic variation among ILs for dry matter partitioning efficiency and seed yield per plant under rainfed conditions, with a subset of ILs exhibiting superior yield performance.

### 3.6 Multivariate ILs and traits relationship

**Figure 6:**
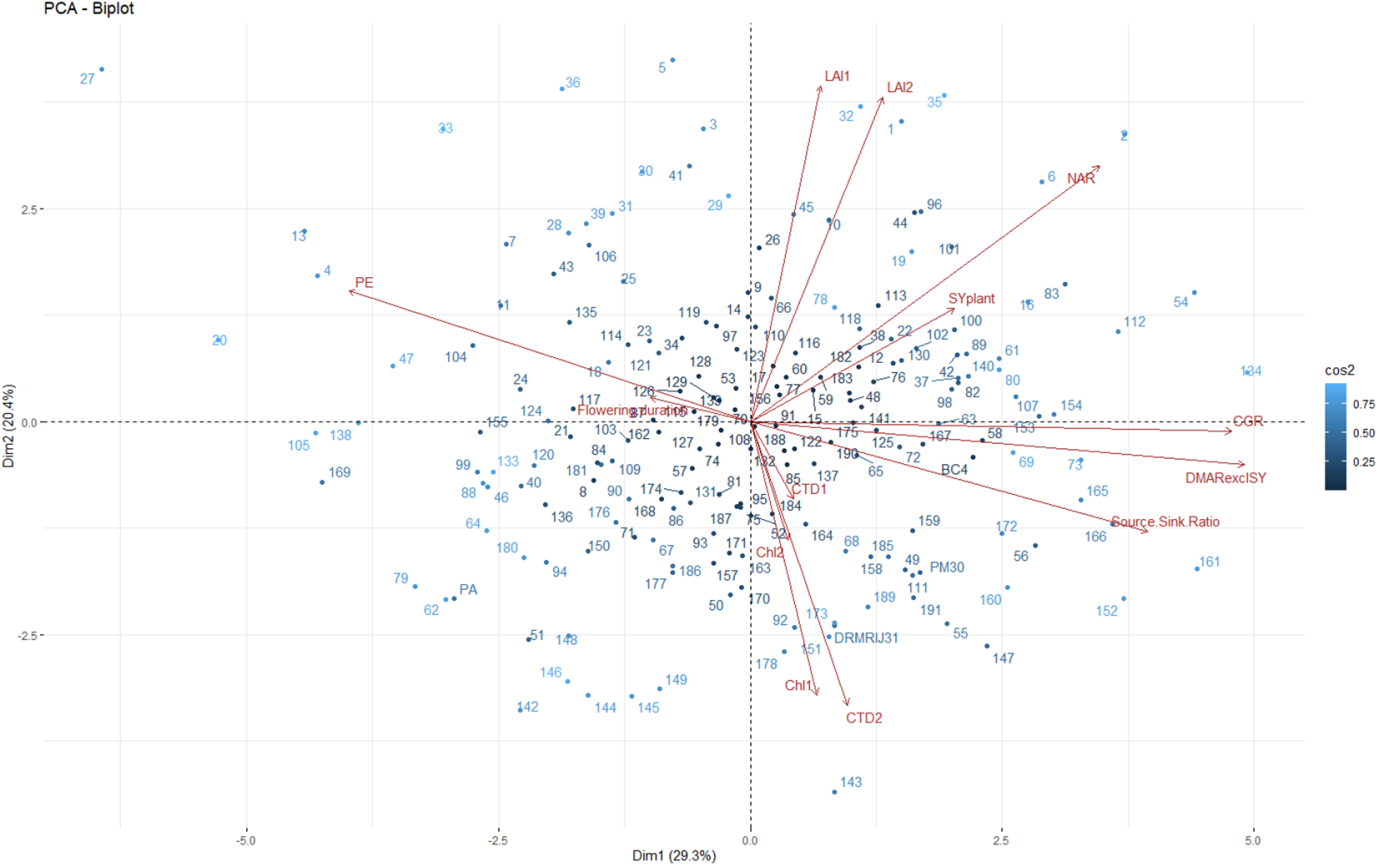
Principal component analysis (PCA) biplot showing the association among introgression lines and key physiological and seed yield-related traits under moisture deficit stress conditions. The first two principal components explain a substantial proportion of total variation, with trait vectors indicating the direction and magnitude of their contribution. The proximity of genotypes to trait vectors reflects their relative performance, while the angles between vectors indicate the strength and nature of correlations among traits.

Principal component analysis (PCA) revealed multivariate relationships among ILs, their parents, and key physiological and seed yield-related traits under rainfed conditions (**Figure 6**). The first two principal components explained a substantial share of the total phenotypic variation, with PC1 accounting for 29.3% and PC2 for 20.4%. The trait vectors in the biplot indicate the direction and magnitude of trait contribution, while the angle between vectors reflects the strength and nature of correlations among traits. Seed yield per plant showed a close association with crop growth rate, dry matter accumulation at the reproductive stage, source–sink ratio, net assimilation rate, and leaf area index at flowering and maturity under moisture-limited conditions (**Figure 6**). The close proximity of these traits in the biplot suggests strong positive associations contributing to yield under moisture deficit conditions. The leaf area index at flowering and maturity was found to be closely associated. Chlorophyll content and canopy temperature depression traits were also observed to be grouped together.

The distribution of ILs across the biplot demonstrated wide genetic variability for source–sink and physiological traits under rainfed conditions. Genotypes located near specific trait vectors indicate higher expression of those traits, whereas those positioned farther away show relatively lower association. Several ILs were consistently positioned near seed yield per plant and major source–sink traits, demonstrating superior performance under moisture-deficit stress conditions. Based on their strong association with multiple yield-related and source–sink traits, IL54, IL83, IL112, IL134, IL161, IL165, and IL73 emerged as the best-performing introgression lines under rainfed conditions. A few ILs, viz., IL56, IL58, IL69, IL153, IL59, IL166, and IL172, exhibited superior performance in terms of crop growth rate, dry matter accumulation at the reproductive stage, and source-sink ratio under moisture deficit stress conditions. In contrast, few ILs were positioned away from yield- and growth-associated traits, indicating comparatively lower association with these traits under rainfed conditions. Overall, the PCA biplot clearly differentiated ILs based on their combined performance for physiological efficiency, source–sink traits, and seed yield under rainfed conditions.

## 4 Discussions

The development and utilization of genetic variability for economically important traits are essential for developing improved *B. juncea* cultivars under adverse climatic conditions (Saroj et al., 2021). Seed yield, an important economic trait, is particularly vulnerable to abiotic stresses such as drought. Within the *Brassica* genus, *B. carinata* exhibits notable tolerance to multiple abiotic stresses, including water deficit stress (Thakur et al., 2019; Limbalkar et al., 2021; Limbalkar et al, 2023). This highlights the importance of transferring genomic regions from *B. carinata* into high-yielding *B. juncea* cultivars to enhance their performance under drought conditions. The present study demonstrates that *B. carinata*-derived *B. juncea* introgression lines exhibit improved coordination between source and sink traits, resulting in enhanced water-use efficiency (WUE) under moisture-deficit conditions. It is important to note that WUE was indirectly measured in this study. The improvement in WUE is likely mediated through better synchronization between photosynthetic assimilate production and reproductive sink demand, which is a key determinant of seed yield stability under drought (Reynolds et al., 2005; Korner, 2015). These findings highlight the effectiveness of exploiting interspecific introgressions to establish a robust source–sink relationship and improve seed yield in *B. juncea* under drought-prone agroecologies.

### 4.1 Genetic variability for agro-morphological and physiological traits

The highly significant differences observed among the ILs for all morphophysiological traits underscore the success of interspecific introgression in broadening the genetic base of *B. juncea*. The ILs not only exhibited variation among themselves but also differed significantly from their parents for almost all traits across environments under rainfed conditions (**Table 1****;** **Supplementary Tables**). This indicates that the introgressed genomic segments from *B. carinata* contributed novel allelic variation without compromising the desirable traits of *B. juncea* (Vasisth et al., 2023). The presence of substantial genetic variability in traits such as dry matter accumulation, flowering time, and seed yield, and its contributing traits, is particularly promising for breeding programs targeting drought resilience. Such variability ensures that superior ILs can be selected for enhanced performance under moisture deficit stress conditions, highlighting the adaptive potential of these lines (Sharma et al., 2018; Singh et al., 2022).

Furthermore, significant genetic variation for all traits under both irrigated and rainfed conditions suggests that these ILs exhibit stable expression of the studied morphophysiological traits across environments, making them reliable candidates for varietal development. The results reinforce the notion that interspecific introgression can be strategically used to combine yield potential with stress adaptability in *B. juncea*, offering a practical route to improve oilseed mustard under adverse climatic conditions. Similarly high magnitude of genetic variability for yield-contributing traits in *Brassica carinata*-derived lines has also been reported earlier, highlighting the effectiveness of interspecific introgression in broadening the genetic base of *Brassica juncea* (Sheikh et al., 2014; Singh et al., 2015; Thakur et al., 2019).

The ILs exhibited substantial phenotypic variability in agromorphological and physiological traits across both rainfed and irrigated environments, as evidenced by wide trait ranges and near-normal frequency distributions (**Figure 1**). Such distribution patterns suggest polygenic regulation of these traits and indicate their suitability for effective phenotypic selection across contrasting moisture regimes. Box–violin plot analysis revealed significant differences in mean performance between rainfed and irrigated conditions for several key traits (**Figure 1**). Collectively, these environmentally driven shifts in morphological, physiological, and biomass-partitioning traits reflect pronounced modulation of source capacity, assimilate translocation, and sink strength in the ILs, thereby underscoring differential source–sink adjustments that contribute to water-use efficiency under moisture-deficit stress conditions. Such adjustments are often associated with maintenance of carbon assimilation and efficient remobilization of stored assimilates to reproductive organs under stress conditions (Blum, 2011; Passioura, 2007).

Notably, higher mean values of 1,000-seed weight, harvest index, chlorophyll content, and CTD2 under rainfed conditions (**Figure 1**) suggested that ILs maintained efficient photosynthetic activity and transpirational cooling despite moisture deficit stress. Maintenance of chlorophyll content and lower canopy temperatures under drought are widely recognized indicators of improved physiological efficiency and stress tolerance in crop plants (Reynolds et al., 2007; Blum, 2011).

In contrast, the absence of significant differences between rainfed and irrigated conditions for traits such as main shoot length, siliqua length, seeds per siliqua, seed yield per plant, plant height at flowering (PH1), CTD1, LAI1, and flowering duration (**Figure 1**) indicates relative stability of reproductive architecture and early canopy development across both water regimes. The significantly higher mean values of most biomass- and growth-related traits under irrigated conditions further emphasize the dependence of assimilate production on moisture availability (Limbalkar et al., 2023).

### 4.2 Association among agro-morphological and physiological traits

Seed yield per plant, the primary sink trait, was strongly governed by coordinated source–sink interactions under both moisture regimes, as evident from correlation and path coefficient analyses (**Figures 2A, B**; **Tables 2**, **3**). Under rainfed conditions, seed yield showed significant positive associations with dry matter accumulation at maturity, crop growth rate, net assimilation rate, harvest index, 1,000-seed weight, leaf area index, and flowering duration, while exhibiting negative correlations with days to 50% flowering and days to maturity (**Figure 2A**). This pattern indicates that early phenology coupled with efficient biomass production and assimilate partitioning is critical for yield realization under moisture deficit stress (Polania et al., 2016; El Sabagh et al., 2019; Geleta et al., 2024). Early-flowering genotypes often escape terminal drought while maintaining sink activity, thereby improving assimilate-use efficiency (Passioura, 2007).

Under irrigated conditions, seed yield per plant showed a positive association with dry matter accumulation at maturity, crop growth rate, net assimilation rate, harvest index, siliqua length, seeds per siliqua, and secondary branches per plant, whereas delayed flowering and maturity exerted negative effects (**Figure 2B**). These results suggest that under irrigated conditions, seed yield is influenced not only by physiological efficiency but also by sink size component traits.

The strong positive correlations of dry matter accumulation at the reproductive stage (excluding seed yield) with crop growth rate, primary and secondary branches per plant, plant height at maturity, days to 50% flowering, days to maturity, net assimilation rate and seed yield per plant under rainfed conditions (**Figure 2A**) highlight the importance of sustained assimilate supply during reproductive growth in ILs. Likewise, net assimilation rate and 1,000-seed weight emerged as key integrative traits linking canopy development, photosynthetic efficiency, and seed yield under moisture-deficit stress (Akanksha et al., 2021; Dubey et al., 2022). These traits collectively reflect the capacity of genotypes to maintain source activity while ensuring effective sink filling under stress conditions.

Path coefficient analysis further refined these relationships by identifying dry matter accumulation at maturity, harvest index, crop growth rate, and net assimilation rate as major direct contributors to seed yield under rainfed conditions (**Table 2**). Although certain traits, such as dry matter accumulation excluding seed yield, showed negative direct effects, their strong positive indirect effects through other traits underscore the complexity of source–sink regulation under moisture-deficit stress in mustard. Under irrigated conditions, dry matter accumulation at maturity, flowering-related traits, crop growth rate, and harvest index exerted the highest direct effects on seed yield, with substantial indirect contributions from reproductive and physiological traits (**Table 3**).

Overall, the combined correlation and path analyses (**Figure 2**; **Tables 2**, **3**) demonstrate that seed yield in *Brassica carinata*-derived *Brassica juncea* introgression lines is primarily driven by efficient biomass production, timely phenology, and effective assimilate partitioning, with moisture regime determining the relative importance of individual traits.

### 4.3 Performance of ILs and parents across environments

The superior performance of ILs across moisture regimes highlights the successful reconfiguration of source–sink relationships for enhanced water-use efficiency. Higher dry matter accumulation at maturity, seed yield, and harvest index in ILs under both rainfed and irrigated conditions indicate enhanced assimilate production and more efficient translocation to reproductive sinks (**Figure 3**), as also evidenced by their superior harvest index. An improved harvest index is widely recognized as an outcome of strengthened sink activity and optimized partitioning rather than increased biomass alone, particularly under water-deficit stress conditions (Foulkes et al., 2011; Blum, 2009). This suggests that ILs possess improved sink strength, enabling greater allocation of assimilates to seeds even under limited water availability.

Comparable crop growth rate and net assimilation rate between ILs and parents under rainfed conditions suggest that introgressed lines can maintain optimal photosynthetic efficiency under moisture-deficit stress, a key component of drought adaptation (Passioura, 2007). In contrast, the marked increase in CGR and NAR of ILs under irrigated conditions (**Figure 3**) indicates greater physiological plasticity and enhanced source capacity when moisture availability is sufficient. Such plastic responses are critical for stabilizing productivity across variable environments and are closely linked to improved water-use efficiency (Condon et al., 2004). Improved water-use efficiency under such conditions is often associated with better coordination between transpiration, carbon assimilation, and biomass partitioning (Condon et al., 2002). The extended flowering duration observed in ILs across environments likely sustained sink demand by prolonging the reproductive phase, thereby facilitating continued assimilate flow to developing mustard siliquae under moisture-deficit stress. Similar associations between longer reproductive phase, improved assimilate remobilization, and yield stability under stress have been reported in *Brassica* and other oilseed crops (Diepenbrock, 2000; Gan et al., 2004). Environment-specific differences in reproductive dry matter accumulation further emphasize differential source–sink regulation, with ILs expressing stronger sink activity under irrigated conditions, while parents showed relatively higher allocation under rainfed conditions (**Figure 3**). Collectively, these results demonstrate that *B. carinata* introgressions have enhanced the balance between assimilate supply and demand, enabling ILs to achieve superior yield and water-use efficiency across moisture regimes.

### 4.4 Relationship between source–sink traits and seed yield

Water-use efficiency (WUE) was not directly measured in this study; therefore, interpretations related to WUE are based on associated morpho-physiological responses under moisture deficit stress conditions. The scatterplot analyses demonstrated substantial variation among introgression lines for both source–sink ratio and seed yield per plant under moisture deficit stress, reflecting differential source–sink regulatory dynamics (**Figure 4**). The overall negative trend between source–sink ratio and seed yield under drought stress suggests that a disproportionately high source relative to sink demand may limit yield formation under drought, consistent with reports that imbalances in source–sink coordination under water deficit can constrain assimilate remobilization and seed filling (Fang et al., 2024). This indicates that optimal rather than excessive source capacity is required, with efficient sink utilization being a critical determinant of yield under stress. Notably, ILs such as IL3, IL11, IL13, IL28, IL33, IL104, IL114, IL124, IL128, IL135, and IL155 clustered near or above the regression trend, indicating efficient source–sink balance and relatively high seed yield even under moisture deficit stress (**Figure 4**). This aligns with findings that genotypes capable of maintaining coordinated assimilate production and sink utilization exhibit higher seed yield resilience under water-deficit stress (Smith et al., 2018).

Under rainfed conditions, dry matter partitioning efficiency was positively correlated with seed yield, indicating that effective allocation of assimilates to reproductive sinks is critical for yield expression under moisture deficit stress (**Figure 5**). ILs, viz., IL11, IL33, IL124, IL128, IL135, IL155, exhibited higher seed yield at moderate partitioning efficiency, underscoring that optimal rather than maximal partitioning supports superior seed yield performance. Such patterns have been reported across crops, underscoring that enhanced partitioning toward seed development increases seed yield under drought by improving assimilate remobilization (Liu et al., 2024). The broad scatter of ILs across both source–sink ratio and partitioning efficiency further highlights the huge genetic variability introduced through *B. carinata* introgressions. These analyses reinforce that balanced source–sink interactions and efficient dry matter partitioning are key determinants of seed yield under moisture deficit stress conditions. Such balance ensures sustained assimilate flow to developing seeds, thereby improving reproductive success under drought stress.

### 4.5 Multivariate relationship among ILs and traits under rainfed conditions

Principal component analysis effectively captured the multivariate relationships among ILs and key physiological and source–sink traits under moisture deficit stress conditions (**Figure 6**). The first two principal components together explained ~50% of the total phenotypic variation, indicating substantial trait-driven differentiation among ILs. The close association of seed yield per plant with crop growth rate, reproductive-stage dry matter accumulation, source–sink ratio, net assimilation rate, and leaf area index at flowering and maturity highlights the integrated contribution of source strength, sink activity, and canopy development towards seed yield under moisture deficit stress conditions (**Figure 6**). Similar trait clustering has been reported for drought-adaptive genotypes that maintain carbon assimilation and assimilate allocation under stress (Passioura, 2007). This further supports that yield under drought is governed by coordinated physiological processes rather than individual traits in isolation.

The grouping of leaf area index at flowering and maturity reflects coordinated canopy development, while the association between chlorophyll content and canopy temperature depression suggests improved photosynthetic stability and transpirational cooling under moisture deficit stress (**Figure 6**), both recognized indicators of drought tolerance and water-use efficiency (Condon et al., 2002). The wide distribution of ILs across the biplot demonstrates selectable genetic variability generated through *B. carinata* introgressions. ILs positioned close to seed yield and major source–sink traits—particularly IL54, IL83, IL112, IL134, IL161, IL165, and IL73—exhibited superior multivariate performance, indicating effective coordination of physiological efficiency and assimilate partitioning under rainfed conditions. Collectively, the PCA results confirm that yield superiority under moisture deficit is governed by the combined expression of source capacity, sink strength, and canopy-level physiological traits rather than by any single trait alone. Such integrative trait expression is essential for achieving stable productivity under water scarce environments.

## 5 Conclusion

Yield performance of *Brassica juncea* introgression lines under moisture-deficit stress was governed by the cumulative contributions of key physiological and source–sink traits rather than by individual yield-component traits. Traits such as crop growth rate, net assimilation rate, leaf area index at flowering and maturity, reproductive-stage dry matter accumulation, harvest index, and optimal source–sink balance collectively contributed to improved seed yield and water-use efficiency under rainfed conditions. Multivariate and trait-association analyses consistently identified IL11, IL33, IL54, IL73, IL83, IL112, IL124, IL128, IL134, IL135, IL155, IL161 and IL165 as superior performers, highlighting their potential as valuable breeding material for drought-prone environments. Integrating source capacity, sink strength, and assimilate partitioning traits in formulating selection indices provides a robust basis for improving drought resilience and yield stability in *Brassica* breeding programs.

## Data Availability

The original contributions presented in the study are included in the article/**Supplementary Material**. Further inquiries can be directed to the corresponding author/s.
