# Supplementary material for: Source-sink relationship in Brassica carinata derived Brassica juncea introgression lines for improving water use efficiency under moisture deficit stress conditions
Source: Front Plant Sci. 2026 May 26;17:1816052. doi: 10.3389/fpls.2026.1816052 (PMC13246383; doi:10.3389/fpls.2026.1816052)
Supplement: Supplementary file 1 [file Table1.docx]

Supplementary Table 1. Analysis of variance of introgression lines (ILs) and parents for different agro-morphological traits evaluated under rainfed (RF) and irrigated (IR) conditions

| Source of Variations |  | Block | | | Treatment | | | ILs | | | Parents | | | ILs vs. Parents | | | Residuals | | | CV (%) | | | |  |
| --- | --- | --- | --- | --- | --- | --- | --- | --- | --- | --- | --- | --- | --- | --- | --- | --- | --- | --- | --- | --- | --- | --- | --- | --- |
| *df* |  | 3 | | | 194 | | | 191 | | | 2 | | | 1 | | | 18 | | |  |  |  |  |  |
|  |  | E1 | E2 | E3 | RE1 | E1 | E2 | E3 | E1 | E2 | E3 | E1 | E2 | E3 | E1 | E2 | E3 | E1 | E2 | | E3 | E1 | E2 | |
|  |  |  |  |  |  |  |  |  |  |  |  |  |  |  |  |  |  |  |  | |  |  |  | |
| PH | RF | 882** | 549.17* | 231ns | 317** | 278.85* | 414** | 293* | 257* | 369** | 1709** | 814** | 3751** | 2107** | 3392** | 2428** | 117 | 136 | 42.2 | | 7.2 | 8.3 | 6.6 | |
|  | IR | 444.0ns | 379.0ns | 59.8ns | 420.0** | 300* | 323* | 376.9* | 272.1* | 316.4* | 814* | 888** | 476ns | 7778** | 4446** | 1264** | 151.3 | 133.8 | 134.7 | | 8.6 | 6.4 | 7.0 | |
| PB/pt | RF | 0.76** | 0.7** | 0.12ns | 0.46** | 0.95** | 0.88** | 0.44** | 0.93** | 0.78** | 0.33** | 1.53** | 9.18** | 2.68** | 2.36** | 3.87** | 0.1 | 0.2 | 0.1 | | 6.4 | 7.9 | 6.0 | |
|  | IR | 7.3** | 0.9ns | 0.8* | 1.95** | 0.78** | 1.3** | 1.9** | 0.76** | 1.25** | 0.73ns | 1.09** | 5.7** | 13.53** | 3.48** | 3.45** | 0.4 | 0.1 | 0.23 | | 11.3 | 6.3 | 7.1 | |
| SB/pt | RF | 3.71* | 0.36ns | 2.7ns | 6.84** | 9.5** | 8.88** | 6.36** | 9.55** | 8.51** | 2.69ns | 0.98ns | 40.2** | 106** | 17.9** | 16.8** | 0.9 | 0.6 | 1.3 | | 9.4 | 7.5 | 10.9 | |
|  | IR | 12.0** | 1.5ns | 4.0** | 8.14.0** | 7.43** | 15** | 7.76** | 7.27** | 14.9** | 0.41ns | 7.28** | 1.6ns | 95.99** | 37.3** | 37.06** | 1.6 | 0.73 | 0.76 | | 11.3 | 7.0 | 6.4 | |
| MSL | RF | 181.7** | 48.4ns | 57.8ns | 107** | 89.96** | 127** | 107.9** | 89.5** | 126** | 91.09ns | 17.2ns | 167.1* | 0.22ns | 330.2** | 196.9* | 33.4 | 26.9 | 31.1 | | 6.7 | 8.8 | 7.3 | |
|  | IR | 257.0** | 104.0ns | 19.0ns | 168** | 99.2** | 127** | 160** | 97** | 127** | 12.8ns | 191.9* | 120ns | 2019** | 323** | 47.91ns | 43.5 | 34.47 | 40.1 | | 7.2 | 6.7 | 8.5 | |
| SoMS | RF | 69.18ns | 25.9ns | 36.8ns | 55.9ns | 44.31** | 52.9** | 49 ns | 44** | 51** | 16.29ns | 65.66* | 210** | 1429** | 111* | 101.3* | 28.9 | 14.9 | 13.5 | | 10 | 8 | 7.2 | |
|  | IR | 25.0* | 43.8ns | 15.2ns | 69.7** | 51.7** | 25.7* | 70.5** | 49.9** | 25.9* | 11.5ns | 150** | 5.9ns | 28.53* | 201** | 23.24ns | 5.8 | 16.45 | 12.3 | | 5.7 | 7.6 | 6.6 | |
| Siliqua length | RF | 0.07** | 0.09ns | 0.06ns | 0.21** | 0.3** | 0.33** | 0.2** | 0.28** | 0.3** | 0.04* | 0.41** | 1.24** | 2.4** | 4.72** | 5.20** | 0.02 | 0.01 | 0.1 | | 5.3 | 4.2 | 5.0 | |
|  | IR | 0.5** | 0.4** | 0.7ns | 0.25** | 0.27** | 0.4** | 0.21** | 0.26** | 0.33** | 0.04ns | 0.52** | 0.22* | 8.38** | 1.04** | 7.20** | 0.04 | 0.04 | 0.05 | | 5.1 | 4.5 | 5.1 | |
| Seeds/ Siliqua | RF | 1.79ns | 0.32ns | 0.24ns | 3.09** | 3.16** | 4.43** | 2.99** | 3.07** | 4.43** | 12.29** | 9.68** | 6.55** | 3.23ns | 5.67** | 0.52ns | 1 | 0.2 | 0.5 | | 7.3 | 4.6 | 4.8 | |
|  | IR | 10.7** | 2.5ns | 2.3ns | 3.02** | 3.65** | 2.7** | 2.86** | 3.5** | 2.7** | 1.17** | 16.1** | 3.11* | 37.82** | 6.05* | 5.94* | 0.1 | 0.9 | 0.83 | | 4.3 | 6.4 | 6.7 | |
| SY/pt | RF | 24.07* | 7.5ns | 5.6ns | 76.8** | 37.17** | 29.6** | 74.87** | 34.6** | 29.3** | 215.8** | 206** | 30.9** | 169** | 191.6** | 78.70** | 6.5 | 4.1 | 2.8 | | 10.6 | 8.1 | 11 | |
|  | IR | 26.0** | 7.0ns | 2.5ns | 71.3** | 44.4** | 40** | 69.3** | 42.9** | 39.8** | 23.2** | 129** | 28** | 552.6** | 170** | 107.0** | 2 | 8.95 | 2.12 | | 6.8 | 11.8 | 8.2 | |
| BY/pt | RF | 609.9** | 114ns | 13ns | 1073** | 466.26** | 369** | 990.6** | 436** | 365** | 6437** | 3503** | 802** | 6053** | 98ns | 161.0** | 72.5 | 46.1 | 13.4 | | 7.5 | 7.0 | 6.6 | |
|  | IR | 537.0** | 53.1ns | 47.1ns | 2026** | 549** | 422** | 1840** | 532** | 426** | 417** | 2150** | 159* | 40716** | 620** | 216.68* | 43.8 | 46.4 | 31.84 | | 6.4 | 6.8 | 7.8 | |
| HI | RF | 3.7ns | 5.56* | 25** | 25.4** | 18.12** | 14.5** | 25.69** | 17.4** | 14** | 5.07ns | 15.9** | 18ns | 12.1ns | 169** | 106.0** | 2.9 | 1.2 | 7.2 | | 8.2 | 6.2 | 9.7 | |
|  | IR | 0.9ns | 3.6ns | 7.0ns | 22.1** | 19.51* | 26** | 22** | 19.5* | 24.5** | 3.57** | 1.39ns | 4.3ns | 88.46** | 57.66* | 404.2** | 0.33 | 7.64 | 3.73 | | 7.4 | 10.8 | 7.9 | |
| SY/plot | RF | 27020  * | 20848  ns | 26193  ** | 92612  ** | 51701  ** | 23945  ** | 89506  ** | 49854  ** | 24082  ** | 348796** | 73037  ** | 5554.2  ns | 173468  ** | 361762  ** | 34525  ** | 7663 | 6738 | 5708 | | 8.9 | 7.9 | 12.2 | |
|  | IR | 45200.0* | 58199.0  ** | 8815.0  ns | 128901  ** | 56564 ** | 42581 ** | 118390  ** | 53650 ** | 40955 ** | 129265  ** | 103840** | 9712  ns | 2135566  ** | 518641  ** | 418942  ** | 11734 | 5254 | 3041 | | 9.6 | 9.0 | 8.3 | |
| TSW | RF | 0.31** | 0.002ns | 0.2ns | 1.07** | 0.67** | 0.88** | 0.86** | 0.54** | 0.67** | 3.86** | 2.8** | 3.86** | 36.8** | 20.7** | 35.10** | 0.02 | 0.1 | 0.1 | | 3.9 | 5.3 | 5.7 | |
|  | IR | 0.4** | 0.1ns | 0.1ns | 0.66** | 0.59** | 0.7** | 0.66** | 0.51** | 0.54** | 0.14** | 4.42** | 2.4** | 1.42** | 9.26** | 19.74** | 0.02 | 0.11 | 0.05 | | 4.5 | 8.3 | 5.1 | |
| OC (%) | RF | 13.5** | 1.85ns | 7.95ns | 7.48** | 4.32** | 8.42** | 7.06** | 4.03** | 8.01** | 15** | 32.9** | 46.1** | 73.1** | 2.4ns | 11.7ns | 1.9 | 0.9 | 3.8 | | 3.9 | 2.6 | 4.6 | |
|  | IR | 2.0ns | 5.4ns | 5.2ns | 595** | 4.53* | 7.71* | 604** | 4.52* | 7.62* | 0.17ns | 5.11ns | 18* | 131.1** | 6.31ns | 4.41ns | 2.71 | 1.79 | 3.79 | | 4.6 | 3.8 | 4.5 | |
| D50%F | RF | 8.22ns | 0.22ns | 19.7ns | 67** | 22.3** | 23** | 60.18** | 19.9** | 21.5** | 244.5** | 126** | 92.2** | 1044** | 279** | 148** | 4.7 | 0.6 | 7.8 | | 4.1 | 3.6 | 5.8 | |
|  | IR | 36.1** | 1.1ns | 8.1ns | 67** | 31.1** | 21** | 60.7** | 28.3** | 19.7** | 3.5ns | 101** | 87** | 1387** | 425** | 81.81** | 1.42 | 0.91 | 2.83 | | 4.1 | 4.9 | 3.2 | |
| DM | RF | 5.78** | 4.17* | 15.3ns | 14.8** | 9.36** | 55.9** | 13** | 8.3** | 49.7** | 193.2** | 98.2** | 429** | 17.9** | 34.17** | 499.0** | 0.3 | 1.2 | 9.1 | | 4.4 | 3.8 | 2.5 | |
|  | IR | 24.4** | 0.9ns | 13.8ns | 22.2** | 4.83** | 56** | 20.2** | 4.3** | 50.7** | 6ns | 45.5** | 301** | 440.0** | 24.3** | 664.1** | 1.91 | 0.57 | 9.65 | | 3.9 | 3.5 | 2.4 | |

*df*= degrees of freedom, E1, E2 and E3 are environments viz., *rabi* season at Delhi (2018-19), Delhi (2020-21) and Bharatpur (2020-21) respectively, PH=Plant height (cm), PB/pt= Primary branches per plant, SB/pt=Secondary branches per plant, MSL=Main shoot length (cm), SoMS=Number of siliquae on main shoot, SY/pt=Seed yield per plant (g), BY/pt= Biological yield per plant (g), HI=Harvest index (%), SY/plot= Seed yield per plot (g), TSW=1,000 seed weight (g), OC= Oil content (%), D50%F=Days to 50% flowering, DM=Days to maturity **Significant at *P*=0.01 and*significant at *P*=0.05, ns= non–significant.

Supplementary Table 2. Analysis of variance of introgression lines (ILs) and their parents evaluated for different physiological traits under rainfed (RF) and irrigated (IR) conditions

| Trait |  | Block | | Treatments | | ILs | | Parents | | | ILs vs. Parents | | Residual | | CV (%) | |
| --- | --- | --- | --- | --- | --- | --- | --- | --- | --- | --- | --- | --- | --- | --- | --- | --- |
| *df* |  | 3 | | 194 | | 191 | | 2 | | | 1 | | 18 | |  |  |
|  |  | E1 | E2 | E1 | E2 | E1 | E2 | E1 | E2 | E1 | | E2 | E1 | E2 | E1 | E2 |
| CC* | RF | 11.5** | 0.17* | 2.44** | 0.08* | 0.12ns | 0.08* | 6.35** | 0.28** | 437.45** | | 0.01ns | 0.75 | 0.04 | 12.34 | 8.67 |
|  | IR | 0.06* | 0.07** | 0.15** | 0.11* | 0.15** | 0.01ns | 0.56** | 0.07** | 0.14** | | 0.03ns | 0.01 | 0.02 | 8.44 | 8.03 |
| CC# | RF | 0.22** | 0.13ns | 0.11** | 0.13** | 0.08** | 0.13** | 2.28** | 0.25* | 0.39** | | 0.11ns | 0.03 | 0.05 | 13.48 | 8.49 |
|  | IR | 0.14** | 0.13** | 0.1** | 0.39** | 0.09** | 0.04ns | 0.11* | 0.13** | 0.95** | | 0.01ns | 0.01 | 0.04 | 8.15 | 7.02 |
| CTD* | RF | 2.08** | 2.27ns | 7.41** | 7.64** | 7.28** | 7.19** | 0.73ns | 1.27ns | 46.81** | | 107.83** | 0.36 | 1.32 | 7.29 | 12.84 |
|  | IR | 2.05** | 4.27** | 4.04** | 2.23** | 3.86** | 69.04** | 46.2** | 3.95** | 0.31ns | | 0.03ns | 0.34 | 0.07 | 11.45 | 9.22 |
| CTD# | RF | 7.57** | 1.23** | 6.11** | 3.78** | 5.2** | 3.83** | 6.02* | 0.77* | 181.31** | | 1.13* | 1.15 | 0.2 | 11.71 | 9.62 |
|  | IR | 2.8** | 4.07* | 3.8** | 0.81ns | 3.56** | 14.31** | 53.62** | 4.05* | 2.14** | | 24.97** | 0.16 | 1.61 | 7.55 | 10.73 |
| LAI* | RF | 2.69** | 0.7ns | 9.4** | 10.44** | 9.47** | 10.52** | 4.96** | 7.53** | 5.78** | | 1.76ns | 0.17 | 0.96 | 6.45 | 14.03 |
|  | IR | 1.25** | 7.43** | 5.16** | 28.77** | 4.92** | 19.6** | 0.04ns | 7.14** | 31.03** | | 1.27ns | 0.14 | 0.91 | 6.74 | 11.06 |
| LAI# | RF | 0.05ns | 15.75** | 18.54** | 12.08** | 14.34** | 11.73** | 2.03** | 39.12** | 852.81** | | 24.97** | 0.07 | 0.53 | 9.34 | 10.59 |
|  | IR | 5.17** | 15.45** | 14.86** | 16.35ns | 13.62** | 139.87** | 235.6** | 14.79** | 22.85** | | 6.12ns | 0.01 | 5.5 | 10.02 | 12.99 |

E1 and E2 are environments viz., *rabi* seasons of 2018-19 and 2020-21 at Delhi respectively, CC= Chlorophyll Content (nmol mg^-1^ fresh weight), CTD= Canopy temperature depression (°C); LAI= leaf area index; * data recorded at 45 days after sowing; # data recorded at 90 days after sowing; E1 and E2 are environments viz., *rabi* season at 2018-19 and 2020-21 at Delhi respectively.
